# Environmental pollutants and the gut microbiota: mechanistic links from exposure to systemic disease

**DOI:** 10.3389/fmicb.2026.1737229

**Published:** 2026-01-23

**Authors:** Wenjing Ma, Xiu Xiong, Zikun Tian, Lan Li, Yi Huang

**Affiliations:** 1 Key Laboratory of Application of Ecology and Environmental Protection in Plateau Wetland of Sichuan, Xichang University, Xichang, Sichuan, China; 2 Key Laboratory of Animal Disease Detection and Prevention in Panxi District, Xichang University, Xichang, China

**Keywords:** dysbiosis, environmental pollutants, gut microbiota, metabolic disorders, oxidative stress

## Abstract

Environmental pollution has emerged as a pervasive global health threat, yet its effects extend far beyond direct organ toxicity. Increasing evidence reveals that the gut microbiota serves as a central mediator of pollutant-induced physiological dysfunctions. This review integrates recent advances on how air pollutants, heavy metals, persistent organic pollutants, and emerging contaminants perturb microbial composition, metabolic activity, and host-microbe signaling. Pollutant exposure alters microbial-derived metabolites such as short-chain fatty acids, bile acids, and tryptophan derivatives, thereby impairing intestinal barrier integrity and immune homeostasis. These microbiota-driven disturbances trigger oxidative stress, chronic inflammation, and neuroendocrine dysregulation, contributing to metabolic disorders, immune imbalance, neurotoxicity, and carcinogenesis. Mechanistically, redox imbalance, activation of TLR4/NF-κB and NLRP3 pathways, and dysregulation of AhR signaling represent critical intersections linking environmental exposure to disease. By elucidating these molecular and ecological connections, this review underscores the gut microbiotaas a key target and therapeutic entry point for mitigating the health impacts of environmental pollution and guiding microbiota-based interventions for disease prevention.

## 1 Introduction

### 1.1 Gut microbiota: a central regulator of human physiology

**Figure 1:**
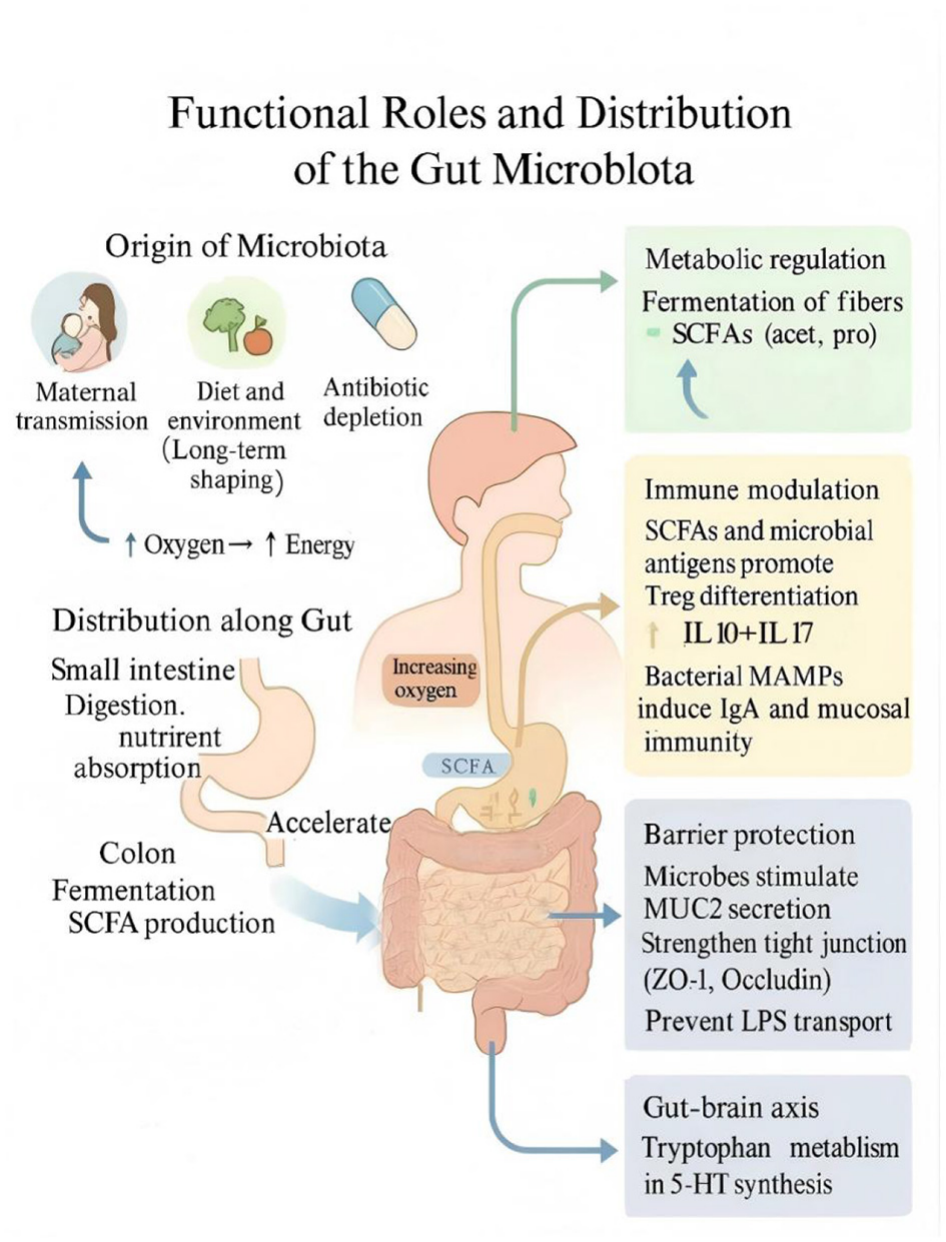
Functional roles and origins of the gut microbiota. The gut microbiota originates from maternal, dietary, and environmental sources and colonizes distinct intestinal niches along the gastrointestinal tract. It performs four fundamental functions essential to host health: (1) metabolic regulation: fermenting dietary fibers to produce short-chain fatty acids (SCFAs) that activate GPR41/43 and AMPK signaling; (2) immune modulation: promoting regulatory T cell (Treg) differentiation, balancing pro- and anti-inflammatory cytokines (IL-10, IL-17), and stimulating IgA secretion; (3) barrier protection: enhancing MUC2 secretion and maintaining tight junction proteins (ZO-1, Occludin, Claudin); and (4) gut-brain communication: regulating tryptophan metabolism and serotonin (5-HT) synthesis, which influence mood and cognition via the vagus nerve. These integrated functions maintain intestinal and systemic homeostasis.

The human intestinal tract harbors an enormous and diverse community of microorganisms, collectively known as the gut microbiota, comprising mainly bacteria, fungi, and viruses. Among them, bacteria dominate, with *Firmicutes, Bacteroidetes*, and *Actinobacteria* being the most abundant phyla (Syromyatnikov et al., 2022). The establishment of gut microbiota is a dynamic and critical process, particularly during early life, which represents a key “window period” for microbial colonization. At birth, the infant's gut microbiota primarily originates from the maternal birth canal and breast milk. As the individual grows, factors such as diet and environment continuously shape the microbial community, leading to a relatively stable structure in adulthood. However, this stability is not absolute and can fluctuate in response to internal or external disturbances (Stewart et al., 2018). The gut microbiota exerts four fundamental functions essential to human health: metabolic regulation, immune modulation, barrier protection, and neurobehavioral communication (Figure 1).

Metabolically, the microbiota assists in the extraction of energy from complex carbohydrates, converting them into absorbable small molecules (Wardman et al., 2022). It also synthesizes short-chain fatty acids (SCFAs) including acetate, propionate, and butyrate, which modulate metabolism and inflammation by binding to specific receptors (such as GPR41 and GPR43) and inhibiting histone deacetylases (HDACs; Sekirov et al., 2010; Jin et al., 2025). Furthermore, the microbiota participates in bile acid metabolism, regulating enterohepatic circulation and bile acid homeostasis. On the other hand, emerging evidence from shotgun metagenomic analyses indicates that pollution exposure is associated with significant alterations in gut microbial composition and inferred microbial functional potential, suggesting pollutant-linked microbial shifts at the gene level. Recent efforts to catalog gut microbial metabolites using combined metagenomic and metabolomic evidence have expanded the known repertoire of microbiota-derived compounds that can enter the host metabolome, underscoring the functional breadth of microbial metabolism (Puig-Castellví et al., 2023). Furthermore, integrative exposome–metabolome perspectives also highlight the broader utility of metabolomics to characterize the biochemical consequences of environmental and microbial influences on human health (Flasch et al., 2022).

In terms of immune modulation, the gut microbiota trains the innate immune system, promoting immune cell development and functional maturation. For instance, it regulates the balance between T helper 17 (Th17) cells and regulatory T (Treg) cells—Th17 cells defend against pathogens, while Treg cells suppress excessive immune responses to maintain homeostasis (Yuan et al., 2023). The microbiota also induces the secretion of immunoglobulin A (IgA), a critical component of mucosal immunity that neutralizes pathogens and harmful antigens (Takeuchi et al., 2021).

For barrier protection, the microbiota maintains the integrity of the intestinal mucus layer, which prevents harmful substances from directly contacting epithelial cells. It upregulates the expression of tight junction proteins such as occludin and claudin, strengthening intercellular connections and preventing paracellular leakage (Xu et al., 2023). Moreover, the microbiota stimulates the production of antimicrobial peptides that directly inhibit pathogenic growth, reinforcing intestinal defense (Natividad et al., 2013).

Through the gut–brain axis, the microbiota engages in bidirectional communication with the central nervous system via neural, immune, endocrine, and metabolic pathways (Carabotti et al., 2015). The vagus nerve serves as a critical conduit for transmitting microbial signals to the brain (Kanai and Teratani, 2022). In addition, the microbiota influences neurotransmitter synthesis and release, including serotonin and dopamine, which are essential for emotion and cognition (Jameson et al., 2025). Immune-mediated signaling also contributes to this communication, as gut microbiota can regulate immune cell activity and thereby affect neuroimmune function (Park et al., 2025).

When microbial homeostasis is disrupted, gut dysbiosis occurs (Fellows et al., 2024). This condition is characterized by reduced microbial diversity (Antharam et al., 2013), depletion of beneficial bacteria such as *Bifidobacterium* and *Lactobacillus* (Zhu et al., 2024), and expansion of opportunistic pathogens that are normally harmless under balanced conditions (Zhao et al., 2023). Functionally, dysbiosis compromises metabolic and immune regulation, increasing the risk of multiple diseases (Liu et al., 2024).

### 1.2 Environmental pollution: a major factor in global health risk

**Figure 2:**
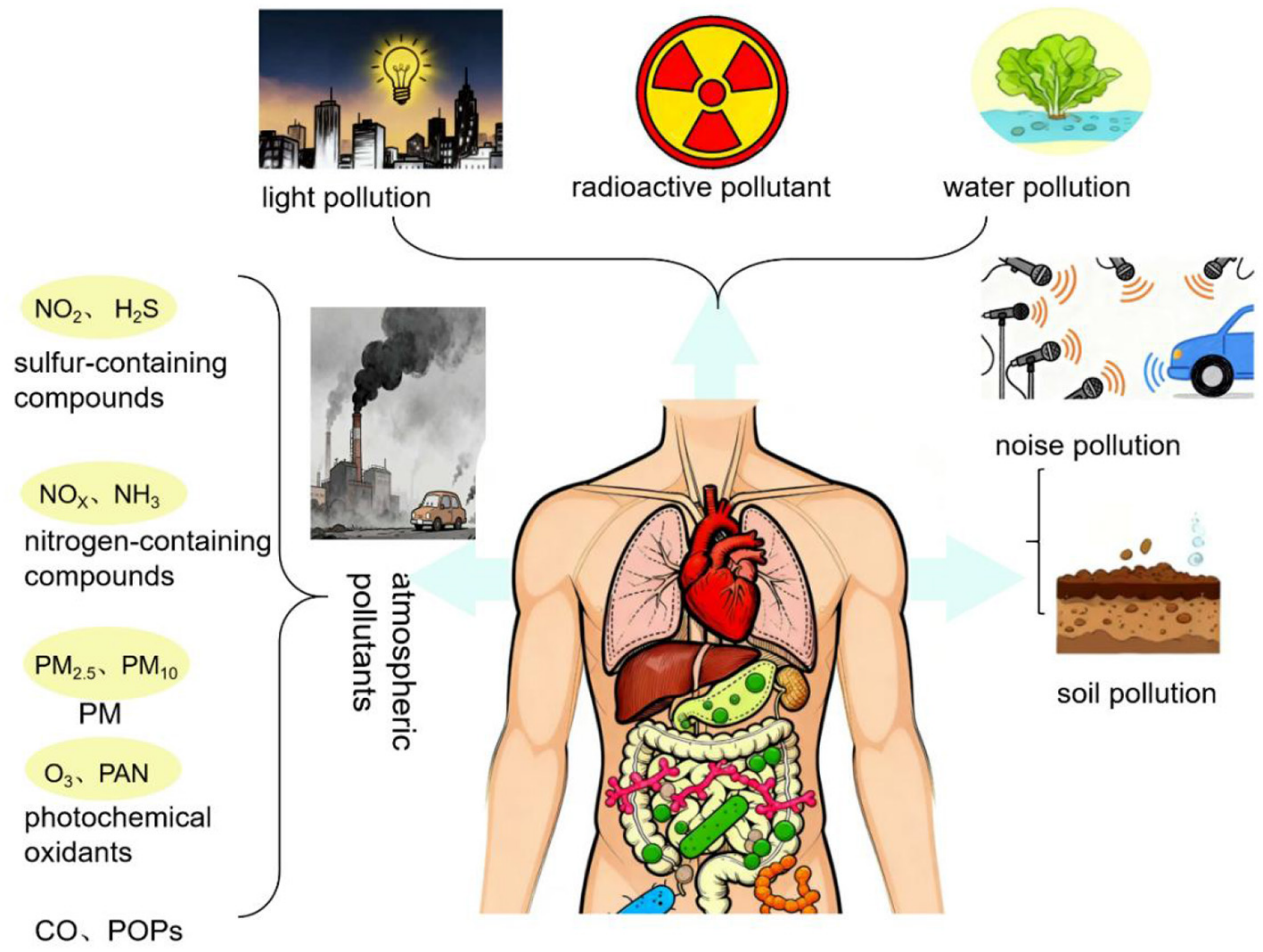
Schematic representation of major environmental pollutants and their entry pathways affecting human health, particularly via gut microbiota. Diverse pollutants including atmospheric (e.g., PM_2.5_, NO_x_, O_3_), waterborne, soil-based, radioactive, light, and noise pollution can influence host physiology. Airborne pollutants such as sulfur-containing compounds (NO_2_, H_2_S), nitrogen-containing gases (NH_3_, NO_x_), photochemical oxidants (O_3_, PAN), particulate matter (PM_2.5_, PM_10_), carbon monoxide (CO), and persistent organic pollutants (POPs) enter primarily through inhalation and systemic absorption. These exposures disrupt multiple organs, particularly the lungs, liver, and gastrointestinal tract, where they may perturb gut microbial homeostasis.

In modern society, environmental pollution has become an insidious yet pervasive threat to human health. A wide variety of pollutants are present in the environment, primarily including air pollutants, heavy metals, persistent organic pollutants (POPs), and emerging contaminants (Figure 2; Aryal et al., 2020).

Air pollutants, such as fine particulate matter (PM_2.5_), ozone (O_3_), and nitrogen dioxide (NO_2_), are distributed unevenly across the globe, with higher concentrations typically observed in industrialized and densely populated regions (Kim et al., 2015). Major sources include industrial emissions, vehicle exhaust, and biomass combustion (Chowdhury et al., 2023). Due to their persistence, air pollutants can remain suspended in the atmosphere for extended periods and undergo long-range transport. The primary route of human exposure is inhalation, allowing these pollutants to enter the respiratory tract and lungs, where they may exert systemic effects through circulation (Chen F. et al., 2024).

Heavy metals, including lead (Pb), mercury (Hg), cadmium (Cd), and arsenic (As), are also widespread in soil and aquatic systems. Their sources encompass mining, industrial production, and agricultural activities, such as the application of metal-containing fertilizers and pesticides (Shang et al., 2023). Owing to their non-degradable nature, heavy metals persist in the environment and bioaccumulate through food chains (Nde et al., 2024). Humans are mainly exposed via ingestion of contaminated crops, drinking water, and aquatic products, as well as dermal contact with polluted soil or water (Ewers, 1990).

Persistent organic pollutants (POPs), such as polychlorinated biphenyls (PCBs), dioxins, and organochlorine pesticides, were once widely used in industrial and agricultural applications. Although many have been banned or restricted, their chemical stability and lipophilicity allow them to persist in ecosystems worldwide (Liu et al., 2016; Negrete-Bolagay et al., 2021). POPs can undergo long-range atmospheric transport, resulting in global contamination (Ochs et al., 2024). Human exposure occurs primarily through dietary intake, especially high-fat foods, but also via inhalation and skin contact (Frederiksen et al., 2009).

Emerging contaminants, such as microplastics and pharmaceutical or personal care products (PPCPs), have recently gained attention as novel environmental threats (Li X. et al., 2024). Microplastics originate from the degradation of plastic waste or industrial production processes and are ubiquitous in marine and freshwater environments (Kushwaha et al., 2024). PPCPs enter the environment primarily through domestic sewage discharge (Jiang et al., 2023). Humans may be exposed to these substances via ingestion of contaminated food (e.g., seafood) or drinking water (Wang Q. et al., 2025). To facilitate a clearer comparison, we have summarized the pollutant categories, typical sources, and primary human exposure routes in Table 1. Compared to traditional pollutants, emerging contaminants are characterized by more recent usage history, a lack of regulatory consensus, and relatively limited evidence regarding long-term microbiota effects. A key feature of these environmental pollutants is chronic low-dose, mixed exposure. Traditional toxicological studies have mainly focused on the direct damage to vital organs such as the lungs, liver, kidneys, and nervous system. However, accumulating evidence indicates that the gut microbiota may also serve as a critical target of environmental pollutants, mediating their systemic effects through microbiota-driven metabolic and immune disturbances.

**Table 1:** Overview of major environmental pollutant categories, representative sources, and exposure pathways.

**Pollutant type**	**Main sources**	**Common exposure routes**
Heavy metals (lead, mercury)	Industrial emissions, heavy metal-containing products (batteries, coatings)	Diet (contaminated food/water), inhalation (heavy metal dust), skin contact
Pesticide residues	Agricultural planting, fruit/vegetable/grain processing	Diet (unwashed fruits/vegetables, contaminated grains)
Plasticizers (phthalates)	Plastic products, food packaging, daily chemical products	Diet (food in contact with packaging), skin contact (daily chemical products)
PM_2.5_	Vehicle exhaust, industrial waste gas, dust	Inhalation (atmospheric intake)
Mycotoxins	Moldy grains, nuts	Diet (consumption of moldy food)
POPs	Legacy industrial/agricultural chemicalss	Diet (fatty foods), inhalation, skin
Emerging contaminants	Plastic degradation, personal care products, sewage	Diet (seafood, water), miexed exposure

However, it is important to note that traditional toxicological frameworks have largely focused on organ-specific outcomes, often overlooking microbiota-mediated mechanisms. This limitation may contribute to inconsistencies across studies and potentially underestimate the true health risks posed by chronic, low-dose exposures. By incorporating gut microbiota as both a target and mediator of pollutant effects, more comprehensive risk assessments and intervention strategies can be developed.

### 1.3 A paradigm shift: the gut microbiota as a new target in environmental toxicology

**Figure 3:**
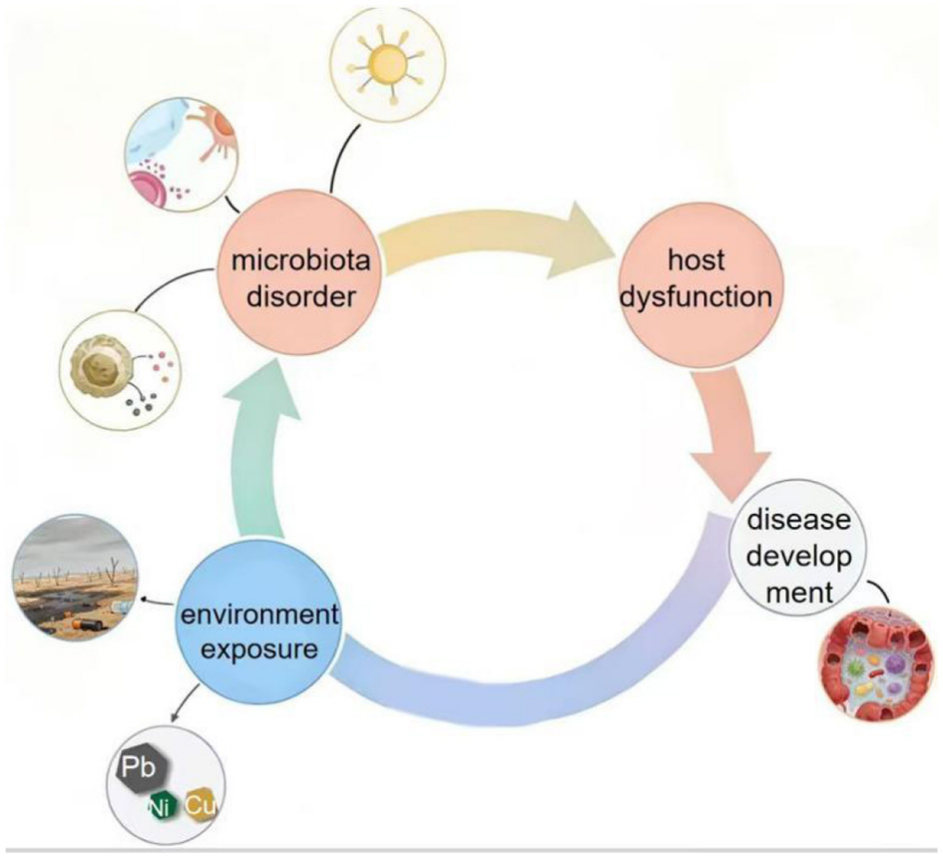
Schematic illustration of the proposed cascade linking environmental pollutant exposure to gut microbiota dysbiosis, host functional disruptions, and chronic disease outcomes. Environmental exposures such as PM_2.5_, lead (Pb), and persistent organic pollutants (POPs) are known to alter gut microbiota composition and function. This dysbiosis, often characterized by decreased short-chain fatty acid (SCFA) production and increased Proteobacteria abundance, may lead to multiple host disruptions including immune activation, metabolic dysfunction, and neuroendocrine imbalance.

Traditional toxicology has primarily focused on the direct cytotoxic effects of environmental pollutants on specific organs such as the liver, kidneys, lungs, and nervous system. However, growing evidence reveals that many pollutants exert indirect yet profound impacts on human health by disrupting the symbiotic gut microbiota (Figure 3). These findings have reshaped the conceptual framework of environmental toxicology, suggesting that the gut microbiota serves not merely as a passive bystander but as an active mediator of pollutant-induced physiological alterations. This paradigm shift raises a critical scientific question: Do environmental pollutants induce host physiological dysfunction and disease through perturbation of the gut microbiota? The aim of this review is to integrate current evidence regarding the bidirectional interactions between environmental pollutants and the gut microbiota, and to construct a mechanistic framework describing the cascade of “environmental exposure → microbiota disruption → host dysfunction → disease development.” This review integrates findings from over 170 peer-reviewed publications, including epidemiological surveys, animal experiments, *in vitro* studies, and multi-omics investigations. By systematically summarizing how various pollutants influence microbial composition and metabolic functions, and how microbial dysbiosis subsequently affects host metabolism, immunity, and neurophysiology, this review seeks to elucidate the underlying mechanisms linking environmental exposure to health outcomes. Furthermore, we discuss the key challenges in this emerging field, including the need for more precise assessment of complex pollutant mixtures, the identification of causal relationships, and the elucidation of molecular interactions between microbes and the host. Finally, future research perspectives are proposed to deepen understanding of the health impacts of environmental pollution and to guide the development of microbiota-targeted strategies for disease prevention and environmental risk mitigation.

## 2 Effects of major environmental pollutants on gut microbiota

To clarify the pollutant selection criteria, we focused on four major categories: air pollutants, heavy metals, persistent organic pollutants (POPs), and emerging contaminants. These groups were chosen based on their global prevalence, well-documented impacts on gut microbiota, and increasing attention from regulatory bodies such as the WHO, USEPA, and national environmental agencies (Aryal et al., 2020). Each category includes representative compounds with substantial evidence of gut microbiota modulation, and their inclusion aims to provide a comprehensive yet focused overview of pollutant–microbiota–host interactions across chemical classes (Table 2).

**Table 2:** Representative pollutants and their effects on gut microbiota, associated mechanisms, and related health outcomes.

**Pollutant category**	**Representative pollutants**	**Gut microbiota effects**	**Key mechanisms involved**	**Related diseases**
Heavy metals	Lead (Pb), cadmium (Cd), mercury (Hg)	SCFA producers (e.g., *Lactobacillus, Bifidobacterium*) ↓; *Proteobacteria* ↑	Oxidative stress, TLR4/NF-κB, epithelial damage	Neurotoxicity, metabolic disorders, inflammation
Particulate matter	PM_2.5_, diesel exhaust particles	α-diversity ↓; proinflammatory taxa (*Desulfovibrio*) ↑	ROS generation, leaky gut, neuroimmune activation	Cognitive decline, depression, asthma
Persistent organic pollutants (POPs)	PCBs, PBDEs, dioxins	Disrupted bile acid metabolism; *Firmicutes*/*Bacteroidetes* ratio ↑	AhR dysregulation, FXR/TGR5 signaling, epigenetic changes	NAFLD, obesity, insulin resistance
Plastic-associated compounds	BPA, phthalates, microplastics	*Akkermansia*, SCFA reduction ↓, LPS-producing bacteria ↑	Barrier disruption, endocrine modulation	Obesity, reproductive and behavioral disorders
Pharmaceuticals and PPCPs	Antibiotics, NSAIDs, SSRIs	Loss of commensals; resistant/opportunistic strains ↑	Dysbiosis, bile salt hydrolase inhibition	Dysbiosis-related inflammation, metabolic effects
Pesticides and herbicides	Glyphosate, chlorpyrifos	Altered microbial diversity and function	EPSPS inhibition, SCFA reduction	Immune suppression, neurodevelopmental toxicity
Metals/metalloids	Arsenic, aluminum	Sulfate-reducing bacteria ↑; beneficial anaerobes ↓	Redox imbalance, methylation/demethylation	Inflammation, carcinogenesis

### 2.1 Air pollutants and their impacts on the gut microbiota

Air pollution is a major global public health concern due to its complex chemical composition and widespread distribution. It comprises various particulate and gaseous pollutants—most notably fine particulate matter (PM_2.5_), ozone (O_3_), and nitrogen oxides (NO_x_)—which can exert both direct and indirect biological effects (Manisalidis et al., 2020). Recent studies have revealed that, beyond causing respiratory and cardiovascular toxicity, air pollutants can influence the gut microbiota through the lung-gut axis, thereby contributing to intestinal dysbiosis and systemic disorders (Yang et al., 2025).

#### 2.1.1 Fine particulate matter (PM_2.5_)

PM_2.5_ refers to airborne particles with an aerodynamic diameter ≤ 2.5 μm, characterized by a large surface area and strong adsorptive capacity for toxic substances such as heavy metals and polycyclic aromatic hydrocarbons. After inhalation, these particles penetrate deep into the alveoli and may translocate via the bloodstream or lymphatic system to distal organs, including the gut, where they significantly alter the microbial ecosystem (Zareba et al., 2024; Dong et al., 2022). Epidemiological and experimental evidence consistently supports a dose- and time-dependent relationship between PM_2.5_ exposure and gut microbial alterations.

In population studies, residents chronically exposed to high PM_2.5_ levels, particularly those living near heavy traffic or industrial areas, show a marked reduction in α-diversity, indicating lower microbial richness and evenness (Li et al., 2023; Shao et al., 2024). Prenatal exposure has also been implicated in adverse pregnancy outcomes; for example, a large cohort study involving 168,852 mothers revealed that maternal PM_2.5_ exposure during gestation was significantly associated with an increased risk of preterm birth (Shi et al., 2024). Animal studies further confirm these findings. Mice exposed chronically to concentrated particulate matter (CPM; 70.9 ± 26.8 μg/m3) exhibited disruption of the intestinal barrier and altered microbiota composition, potentially mediated through activation of the TLR2/5–MyD88–NLRP3 signaling axis (Ran et al., 2024). Another experiment exposing mice to 198.5 μg/m3 PM_2.5_ resulted in transient weight loss followed by compensatory gain, accompanied by marked alterations in intestinal microenvironment and metabolic signaling pathways (Dai et al., 2022).

Mechanistic insights have been provided by Shao et al. (2024) using a Versatile Aerosol Concentration Enrichment System (VACES) to expose male C57BL/6J mice to concentrated ambient PM_2.5_ (CAP) or filtered air (FA). Through antibiotic-induced microbiota depletion and fecal microbiota transplantation (FMT), the study demonstrated that PM_2.5_-induced glucose metabolic disorders were mediated by gut microbiota imbalance. Further analysis identified changes in short-chain fatty acids (SCFAs), particularly acetate, as a key metabolic link, and acetate supplementation effectively ameliorated the metabolic abnormalities (Shao et al., 2024). Together, these findings suggest that the gut microbiota plays a critical mediating role in PM_2.5_-related metabolic dysfunction.

#### 2.1.2 Ozone (O_3_) and nitrogen oxides (NO_*x*_)

Ozone (O_3_), a triatomic oxygen molecule with strong oxidative capacity, exists in both the stratosphere (as the protective “ozone layer”) and the troposphere, where it acts as a major pollutant (Chen et al., 2020). Ground-level ozone has become one of the most serious environmental issues in China and other rapidly developing regions (Feng et al., 2015). Its impact on the gut microbiota is primarily mediated through oxidative stress and systemic inflammation. For example, Ramot et al. (2015) exposed healthy and cardiovascular disease–prone rats to various concentrations of ozone for 4 h and observed species-specific differences in pulmonary and renal inflammation. Although no direct gut injury was reported, systemic inflammatory responses were evident, suggesting a potential link to gut microbial alterations through immune–oxidative pathways (Ramot et al., 2015).

Nitrogen oxides (NO_x_), including nitric oxide (NO) and nitrogen dioxide (NO_2_), are another class of traffic-related air pollutants with emerging relevance to gut microbiota disturbances (Leclerc et al., 2021; Sayegh et al., 2016). Epidemiological data show that exposure to traffic-related air pollution (TRAP) correlates with both gut microbiome composition and fasting blood glucose levels. Specifically, TRAP exposure is associated with a decrease in *Bacteroidaceae* abundance (*r* = −0.48, *p* = 0.001) and an increase in *Ruminococcaceae* (*r* = 0.48, *p* < 0.001). Furthermore, *Bacteroidaceae* abundance negatively correlates with fasting glucose (*r* = −0.34, *p* = 0.04), while *Ruminococcaceae* shows a positive correlation (*r* = 0.41, *p* < 0.01). Path analysis revealed that changes in these two microbial taxa explained approximately 24%−29% of the association between TRAP exposure and elevated fasting glucose levels (Alderete et al., 2018). These findings indicate that air pollution may influence host glucose metabolism via microbiota-mediated pathways.

### 2.2 Effects of heavy metals on the gut microbiota

Heavy metals are a class of persistent and bioaccumulative environmental contaminants characterized by their strong toxicity and inability to be degraded. Human exposure occurs primarily through ingestion of contaminated food and drinking water, as well as through occupational and environmental contact. Chronic exposure has been associated with carcinogenesis, DNA damage, and irreversible impairment of the immune system (Valko et al., 2006).

Recent studies have revealed that heavy metals also exert toxicity by directly interacting with the gut microbiota and intestinal barrier, reshaping microbial community structure through selective pressure, oxidative stress, and gene regulation, and thereby disrupting microbial functions (Breton et al., 2013; Liu et al., 2021). Importantly, different heavy metals display distinct modes of action and biological consequences.

#### 2.2.1 Arsenic (As)

Arsenic exists in both inorganic (As^3+^, As^5+^) and organic forms (e.g., monomethyl and dimethyl arsenic), with inorganic arsenic being considerably more toxic (Ganie et al., 2024). Drinking water is the major exposure route for humans, particularly in endemic regions such as Bangladesh and parts of western China, where arsenic concentrations in groundwater often exceed 10 μg/L (Sadee et al., 2025). Epidemiological studies have shown that high arsenic exposure correlates with distinct alterations in gut microbial composition. For example, children from arsenic-contaminated areas exhibited higher abundances of *Proteobacteria* and enrichment of 332 SEED functional genes related to virulence and antibiotic resistance. Moreover, *Escherichia coli* strains isolated from these children carried unique arsenic resistance operons with increased gene expression levels (Dong et al., 2017).

Animal experiments further confirmed the impact of arsenic on the gut microbiota. In a long-term study, C57BL/6 mice exposed to 0, 5, or 10 ppm sodium arsenite (NaAsO_2_) via drinking water for 6 months developed significant liver injury and microbial dysbiosis (Li H. et al., 2024). Using 16S rRNA gene sequencing, Lu et al. (2014) demonstrated that 4 weeks of exposure to 10 ppm inorganic arsenic profoundly altered the gut microbial community structure in C57BL/6 mice.

Metabolomic profiling further revealed that arsenic exposure caused widespread disturbances in metabolites across multiple biological matrices (blood, liver, feces), many of which were products of microbial metabolism, indicating a close link between microbial dysfunction and systemic metabolic imbalance (Lu et al., 2014).

#### 2.2.2 Cadmium (Cd) and Lead (Pb)

Cadmium and lead are among the most prevalent environmental heavy metals and share similar mechanisms of gut toxicity. Both induce oxidative stress, promote the selection of resistant bacterial strains, and disrupt microbial homeostasis (Liu et al., 2023).

Cadmium contamination primarily occurs through dietary intake, especially from rice cultivated in polluted soils (Wang et al., 2019). In a study by Wang H. et al. (2025), mice exposed to 3 mg/L cadmium in drinking water for 9 weeks exhibited cognitive impairment secondary to gut dysbiosis. The exposure disrupted the intestinal barrier, altered inflammatory cytokines, affected hippocampal gene expression, and modified gut-derived neuroactive metabolites, providing evidence that cadmium neurotoxicity is mediated through the gut–brain axis, with the microbiota serving as a key target for prevention and intervention (Wang H. et al., 2025). Similarly, studies on the freshwater snail *Cipangopaludina chinensis* revealed that cadmium exposure inhibited respiratory metabolism and immune responses, induced oxidative stress, and upregulated genes related to energy metabolism, suggesting an adaptive response to chronic cadmium stress (Wu et al., 2023).

Lead exposure, comparable in pathway to cadmium, occurs mainly through contaminated food and water (Kim et al., 2014). A population study involving 696 participants demonstrated that urinary lead concentration was positively associated with both α-diversity and β-diversity shifts in the gut microbiota. Specifically, higher urinary lead levels correlated with increased abundance of *Proteobacteria* and *Burkholderiales*, indicating that lead exposure may promote the proliferation of opportunistic taxa capable of heavy-metal tolerance (Eggers et al., 2019).

#### 2.2.3 Mercury (Hg)

Mercury can be converted in the environment into methylmercury (MeHg), a highly toxic and bioaccumulative compound that enters the human body primarily through consumption of fish, particularly large predatory species (Wang et al., 2020a; Nielsen et al., 2018). Experimental data from multiple fish species suggest that dietary MeHg exposure rarely causes acute lethality, but it significantly affects growth, reproduction, and behavior at concentrations above 0.2–0.5 μg/g wet weight (Depew et al., 2012). Field observations confirm that even low MeHg levels may disrupt population stability in wild fish, though relevant ecological studies remain limited. In humans and mammals, both inorganic divalent mercury (Hg^2+^) and MeHg induce neurotoxicity and immunotoxicity, mainly through gastrointestinal exposure. However, the direct effects on intestinal epithelium and microbiota have been less studied. Using the Caco-2 intestinal epithelial cell model, researchers demonstrated that exposure to 0.5–1 mg/L of Hg^2+^ or MeHg (comparable to concentrations found in contaminated food) disrupted redox homeostasis, increased cellular permeability, and damaged tight junction integrity (Vázquez et al., 2014). These findings suggest that mercury exposure compromises intestinal barrier function, providing an entry point for systemic toxicity.

Beyond compositional changes, gut microbiota play an active role in the biotransformation of heavy metals through enzymatic and redox-mediated processes. For instance, specific microbial taxa can methylate or demethylate mercury and arsenic, thereby altering their chemical speciation, toxicity, and bioavailability. Sulfate-reducing bacteria have been shown to convert inorganic mercury into methylmercury, a neurotoxic form with enhanced absorption potential (Tepper et al., 2025). Conversely, demethylation or sequestration pathways may mitigate toxicity. Additionally, microbial redox transformation of metals such as chromium (Cr) and selenium (Se) can alter their valence state and associated risks (Raab, 2003). These transformations are mediated by microbial genes encoding metal reductases, oxidases, and transporters, which may be enriched in the gut microbiome under chronic exposure scenarios. Thus, microbial biotransformation serves as a double-edged sword—modulating, amplifying, or detoxifying metal species within the gut ecosystem.

### 2.3 Effects of persistent organic pollutants (POPs) on the gut microbiota

Persistent organic pollutants (POPs) are highly toxic, lipophilic, and environmentally stable chemicals characterized by long-range transport capability and strong bioaccumulation potential (Aravind Kumar et al., 2022). Major categories include polychlorinated biphenyls (PCBs), dioxins (e.g., TCDD), organochlorine pesticides (e.g., DDT), and polybrominated diphenyl ethers (PBDEs; Choo et al., 2020). These compounds can bioaccumulate through food chains and pose significant ecological and health risks.

As research on the gut microbiota has advanced, it has become increasingly evident that the microbiota serves not only as a target of POP toxicity but also as an active mediator of pollutant metabolism and host response (Lee et al., 2024; Hou et al., 2025). Recent findings suggest that POP exposure disrupts gut microbial homeostasis, thereby contributing to metabolic, immune, and neurobehavioral disorders (Popli et al., 2022).

#### 2.3.1 Polychlorinated biphenyls (PCBs) and dioxins (TCDD)

PCBs and TCDD are classic ligands of the aryl hydrocarbon receptor (AhR), a transcription factor that regulates xenobiotic metabolism and immune responses. Activation of AhR by these pollutants can alter both host and microbial gene expression, leading to dysbiosis and impaired intestinal function. PCBs, a class of chlorinated aromatic compounds, remain widespread in the environment despite decades of regulation (Kimbrough, 1995). Experimental studies have shown that maternal exposure to PCB-126 significantly reduces microbial richness and diversity in adult offspring, alters specific bacterial taxa, and potentially increases susceptibility to chronic diseases later in life—effects that appear independent of diet or physical activity (Agarwal et al., 2023). Mechanistically, PCB-induced AhR activation enhances the expression of proinflammatory cytokines such as IL-6 and TNF-α, creating a chronic inflammatory microenvironment that suppresses the growth of beneficial, inflammation-sensitive bacteria (Lee et al., 2015).

TCDD (2,3,7,8-tetrachlorodibenzo-p-dioxin), one of the most toxic dioxin congeners, exerts similar effects through sustained AhR activation (Garattini, 1982). In mice, TCDD exposure has been shown to alter bile acid metabolism and induce non-alcoholic fatty liver disease (NAFLD), accompanied by increases in secondary bile acids and specific bacterial taxa such as Lactobacillus. Metagenomic analysis further revealed changes in genes associated with bile acid synthesis, suggesting a gut–liver axis–mediated mechanism (Fling and Zacharewski, 2021). In another study, Li J. et al. (2022) investigated maternal and lactational TCDD exposure in mice and found that high-dose exposure led to both maternal and offspring dysbiosis, disrupted tryptophan metabolism, and increased abundance of pathogenic bacteria. Interestingly, low-dose exposure had partial protective effects, reducing pathogenic taxa in offspring, indicating dose-dependent and transgenerational impacts of TCDD on gut microbial ecology (Li J. et al., 2022).

#### 2.3.2 Organochlorine pesticides (DDT) and brominated flame retardants (PBDEs)

**Figure 4:**
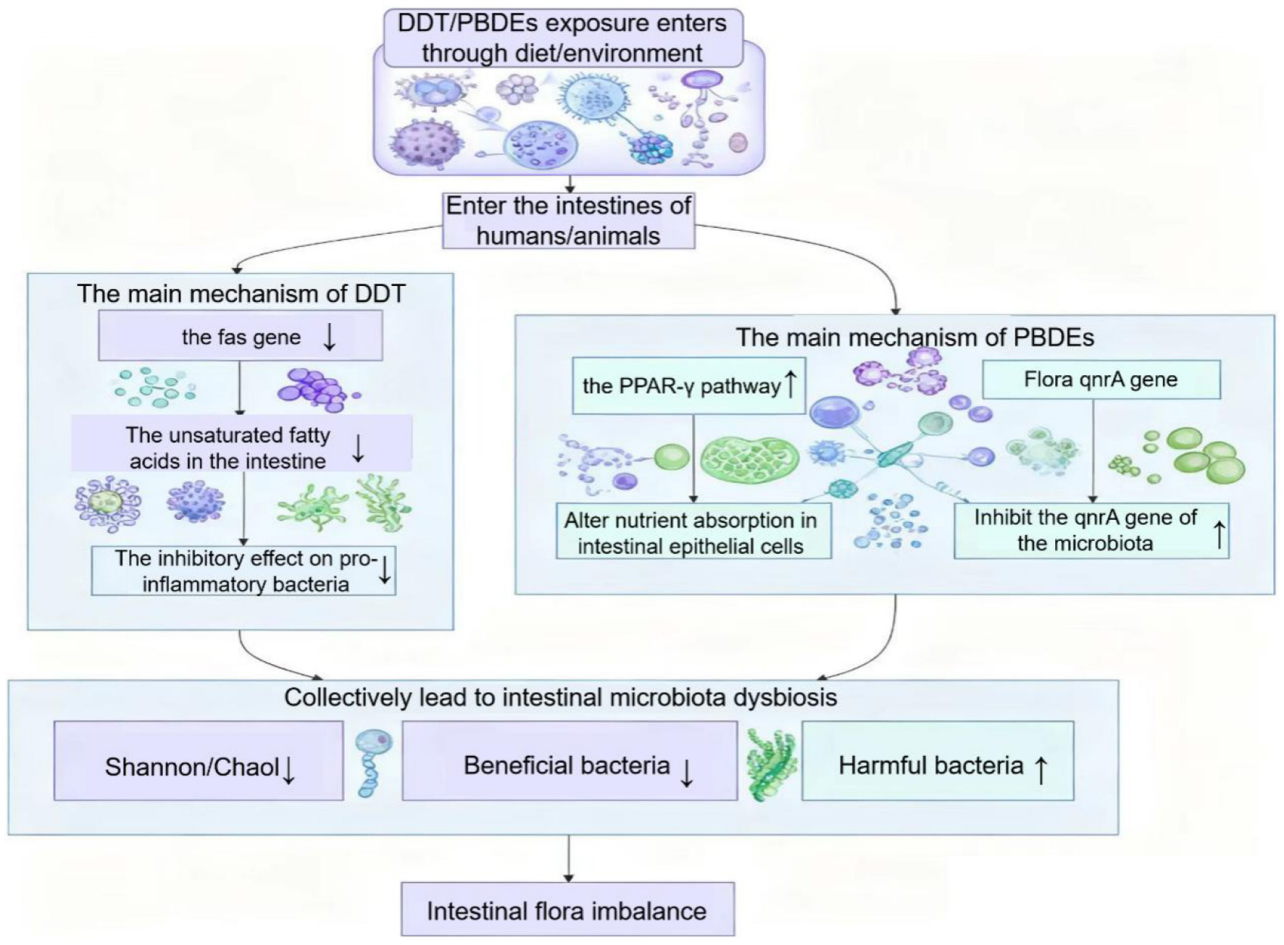
Pathways by which DDT and PBDEs cause intestinal microbiota dysbiosis. This schematic illustrates how environmental exposure to dichlorodiphenyltrichloroethane (DDT) and polybrominated diphenyl ethers (PBDEs), primarily via dietary intake and environmental contact, leads to microbial dysbiosis. DDT disrupts the fas gene expression and reduces unsaturated fatty acids in the intestine, thereby weakening the suppression of pro-inflammatory bacteria. PBDEs activate the PPAR-γ pathway and inhibit the microbial qnrA gene, altering nutrient absorption in epithelial cells and microbial gene regulation. These disturbances collectively reduce microbial diversity (e.g., Shannon and Chao1 indices), decrease beneficial bacterial populations, and increase harmful taxa, ultimately contributing to intestinal flora imbalance.

Dichlorodiphenyltrichloroethane (DDT), a well-known organochlorine pesticide, has been banned in most countries but remains persistent in the environment due to its high stability (Sudharshan et al., 2012). PBDEs, commonly used as flame retardants in plastics, textiles, and electronics, are another group of widespread POPs with structural and toxicological similarity to PCBs (Jiang et al., 2024). Both can alter gut microbial communities, though their specific effects differ depending on exposure conditions (Figure 4).

A study examining perinatal PBDE-47 exposure found that it disrupted gut microbiota development, growth, and metabolism in offspring. However, maternal supplementation with Lactobacillus reuteri during pregnancy and lactation mitigated these effects in a sex-dependent manner, restoring microbial diversity and improving body weight and neurobehavioral performance in offspring (Denys et al., 2025). Similarly, Tian et al. (2014) explored short-term PBDE-47 exposure in the marine sponge *Cymaeformis* and observed a time- and dose-dependent shift in the bacterial community from autotrophic to heterotrophic dominance, with loss of sulfur-oxidizing symbionts and enrichment of heterotrophic bacteria harboring specific metabolic genes. These results indicate that PBDEs exert selective pressure on microbial populations, reshaping both composition and functional potential.

### 2.4 Emerging pollutants and their effects on the gut microbiota

With advances in environmental detection technologies, a new class of pollutants collectively referred to as emerging contaminants has gained increasing attention. Among them, microplastics (MPs), nanoplastics (NPs), antibiotic resistance genes (ARGs) and phamaceuticals and personal care products (PPCPs) represent major concerns due to their persistence, ubiquity, and potential to disrupt host-microbe interactions. These pollutants enter the human body primarily through food, drinking water, and environmental contact, and they can induce intestinal toxicity via physical damage, chemical leaching, and genetic transfer mechanisms. Their health implications have become an emerging research frontier in environmental toxicology.

#### 2.4.1 Microplastics and nanoplastics

Microplastics (MPs, 1 μm to 5 mm) and nanoplastics (NPs, <1 μm) are small plastic fragments produced through environmental degradation or industrial processes. They are widespread in aquatic, terrestrial, and atmospheric systems (Thompson et al., 2004). The impacts of these particles on gut microbiota involve three interrelated mechanisms: physical stress, chemical toxicity, and carrier (vector) effects (Qiao et al., 2021; Hsu et al., 2025). The magnitude of toxicity depends on particle size, surface chemistry, and polymer type.

##### 2.4.1.1 Physical effects

Smaller particles exhibit greater biological reactivity. Experimental studies have shown that ingestion of nanosized plastics causes more pronounced intestinal and hematopoietic toxicity than larger particles (Zhang et al., 2023; Jing et al., 2022). For instance, Jing et al. (2022) demonstrated that oral exposure of mice to micro/nanoplastics (MNPLs) induced hematopoietic toxicity and gut dysbiosis, and these alterations were closely correlated with the observed blood toxicity. Mechanistically, NPs can penetrate epithelial tight junctions and reach the mucosal layer, where they directly contact gut microorganisms. This physical friction damages bacterial membranes, increases permeability, and triggers microbial apoptosis (Hu et al., 2022). In mice, oral exposure to 2 μm polyvinyl chloride (PVC) MPs caused intestinal injury and altered both microbiota composition and metabolite profiles, suggesting potential human health risks through chronic low-dose exposure (Chen X. et al., 2022).

##### 2.4.1.2 Chemical effects

Toxicity can also result from the leaching of plastic additives, such as phthalates (e.g., di-(2-ethylhexyl) phthalate, DEHP), which are commonly used as plasticizers (Meeker et al., 2009). Yu et al. (2021) reported that long-term DEHP exposure (0.5 mg/kg/day for 23 weeks) led to cholesterol imbalance in rats. Fecal microbiota transplantation and 16S rRNA sequencing revealed that DEHP disrupted gut microbial composition, altered the bile acid profile, and activated intestinal farnesoid X receptor (FXR) signaling, thereby suppressing hepatic bile acid synthesis. These results indicate that microplastic-associated chemicals may modulate bile acid metabolism via the gut–liver axis (Yu et al., 2021). In addition, plastic polymer type affects toxicity: polystyrene (PS) particles exhibit higher affinity for adsorbing toxic substances than polyethylene (PE) or polyethylene terephthalate (PET), leading to stronger microbial disturbances (Lithner et al., 2012).

##### 2.4.1.3 Carrier (vector) effects

A unique feature of MPs and NPs is their ability to act as vectors that adsorb and transport other environmental pollutants, including heavy metals and POPs, into the gut (Ortega and Cortés-Arriagada, 2023). For example, Deng et al. (2020) demonstrated that MPs could adsorb phthalates (PAEs) and deliver them into the mouse intestine. The intestinal accumulation order of PAEs (DEHP > DBP > DEP > DMP) corresponded with their adsorption affinity on MPs. After 30 days of combined exposure to DEHP-contaminated MPs, mice showed significantly increased intestinal permeability, aggravated inflammation, and altered microbiota composition, particularly affecting bacteria involved in energy metabolism and immunity. Transcriptomic analysis identified 703 differentially expressed genes related to oxidative stress and inflammation (Deng et al., 2020). These findings highlight that MPs–additive complexes can exert synergistic toxicity, emphasizing the complexity of real-world exposure scenarios.

#### 2.4.2 Antibiotic resistance genes (ARGs)

Antibiotic resistance genes (ARGs) are mobile genetic elements that confer antibiotic resistance to microorganisms and are now recognized as a novel class of environmental contaminants (Zhao Y. et al., 2025). ARGs are abundant in soil, water, and animal waste, and can enter the human gut through consumption of contaminated food (e.g., vegetables, meat) or drinking water (Tan et al., 2023). Once inside the gut, ARGs can be horizontally transferred into commensal or pathogenic bacteria via horizontal gene transfer (HGT) mechanisms, leading to an expanded intestinal “resistome” and heightened risk of antibiotic-resistant infections (Haug et al., 2011).

Current studies focus on the efficiency of ARG transfer and its influence on gut microbiota composition (Casals-Pascual et al., 2018). HGT in the gut microbiome commonly occurs via transduction and conjugation, which facilitate gene exchange between symbiotic and opportunistic bacteria. Novel bioinformatic tools have enabled the identification of specific ARG–host associations and quantification of HGT frequency within microbial communities, offering new insights for developing targeted interventions to limit resistance gene propagation (McInnes et al., 2020).

Animal experiments further confirm the ecological risks of dietary ARG exposure. Using high-throughput quantitative PCR and 16S rRNA sequencing, researchers compared mice fed organically and conventionally grown lettuce and wheat for 8 weeks. In the organic group, the abundance and diversity of ARGs (e.g., tetracycline and multidrug resistance genes), mobile genetic elements (MGEs), and potential antibiotic-resistant bacteria (ARBs) increased significantly over time, whereas no such trend was observed in the conventional group. Additionally, MGEs such as IS613 were shown to modulate ARG profiles, while resistant pathogens including Bacteroides and Streptococcus became more enriched (Zhuang et al., 2024). This suggests that organic produce may serve as an unrecognized vector for ARG dissemination in the gut microbiota, underscoring the need for comprehensive risk assessment. The proliferation of resistant bacteria increases the likelihood of clinical treatment failure, forming a vicious cycle: from environmental ARGs to intestinal resistant bacteria to therapeutic resistance, which poses a major challenge to public health management (Yuan et al., 2024).

#### 2.4.3 Phamaceuticals and personal care products (PPCPs)

Pharmaceuticals and personal care products (PPCPs), encompassing antibiotics, analgesics, antidepressants, hormones, antiseptics, UV filters, and cosmetic ingredients, are an increasingly prominent class of environmental pollutants. They enter the environment primarily through wastewater discharge and agricultural runoff and are often resistant to complete degradation in conventional treatment systems (Jiang et al., 2023). As such, PPCPs represent a chronic, low-dose exposure risk to both aquatic organisms and humans.

Mounting evidence suggests that PPCPs can disrupt gut microbiota composition, diversity, and function, even at environmentally relevant concentrations. For instance, triclosan, a widely used antimicrobial found in soaps and toothpaste, has been shown to reduce the relative abundance of butyrate-producing *Lachnospiraceae* and *Ruminococcaceae* while increasing pro-inflammatory taxa such as *Proteobacteria* (Sinicropi et al., 2022). In mice, chronic triclosan exposure altered microbial metabolic pathways, leading to impaired epithelial barrier function and exacerbated colitis (Lian et al., 2025).

Non-antibiotic PPCPs such as carbamazepine (an antiepileptic) and fluoxetine (an antidepressant) have been shown to modulate microbial gene expression related to neurotransmitter metabolism and stress responses. In zebrafish models, fluoxetine exposure led to increased abundance of *Actinobacteria* and shifts in amino acid biosynthesis pathways, potentially linking microbiota changes to neurobehavioral phenotypes (Pinto et al., 2024). Moreover, metagenomic and metabolomic profiling of human populations exposed to multiple PPCPs (including NSAIDs and UV filters) revealed altered bile acid metabolism and reduced short-chain fatty acid (SCFA) levels, with implications for systemic inflammation and metabolic syndrome (Cheng et al., 2025).

Despite these advances, knowledge gaps remain regarding long-term consequences, mixture effects, and inter-individual variability in microbiota responses. Notably, most functional studies to date are limited to *in vivo* rodent models or aquatic species, with fewer high-resolution, longitudinal studies in human cohorts (Jiang et al., 2023). Future research should prioritize integrating multi-omics approaches, such as metagenomics, metabolomics, and transcriptomics with environmental exposure assessment to unravel the causal pathways linking PPCPs to host-microbiota dysregulation.

Taken together, these findings underscore the complex and dynamic nature of pollutant-induced gut microbiota disturbances. While short-term exposures often lead to reversible or transient microbial changes, longer-term exposures, particularly in animal models, have been shown to induce persistent shifts in microbiota composition and function. However, in humans, evidence for the persistence of such changes remains scarce, and further longitudinal and interventional studies are warranted to better elucidate the temporal stability of pollutant-driven dysbiosis.

## 3 Mechanistic insights: from molecular pathways to systemic effects

Pollutant-induced gut microbiota alterations may result from both direct and indirect mechanisms. Direct microbial toxicity includes oxidative stress, membrane damage, genotoxicity, and disruption of quorum sensing, especially relevant for heavy metals, antibiotics, and nanoparticles. In parallel, pollutants can elicit host metabolic, endocrine, and immunological responses that secondarily influence microbial composition. For example, perfluorinated compounds can alter bile acid metabolism and immune tone, indirectly reshaping gut microbial communities (Zhao M. et al., 2025). These primary interactions may further engage well-characterized mechanistic pathways, including oxidative stress responses, activation of the aryl hydrocarbon receptor (AhR) signaling axis, impairment of intestinal epithelial barrier integrity, and amplification of mucosal inflammation. Such pathways may not only mediate microbiota shifts but also contribute to systemic health consequences. In the sections that follow, we review current evidence for these core mechanisms, highlighting their interconnections and implications for host-microbiota dynamics under environmental pollutant exposure.

### 3.1 The central mediating role of oxidative stress in microbiota disruption

**Figure 5:**
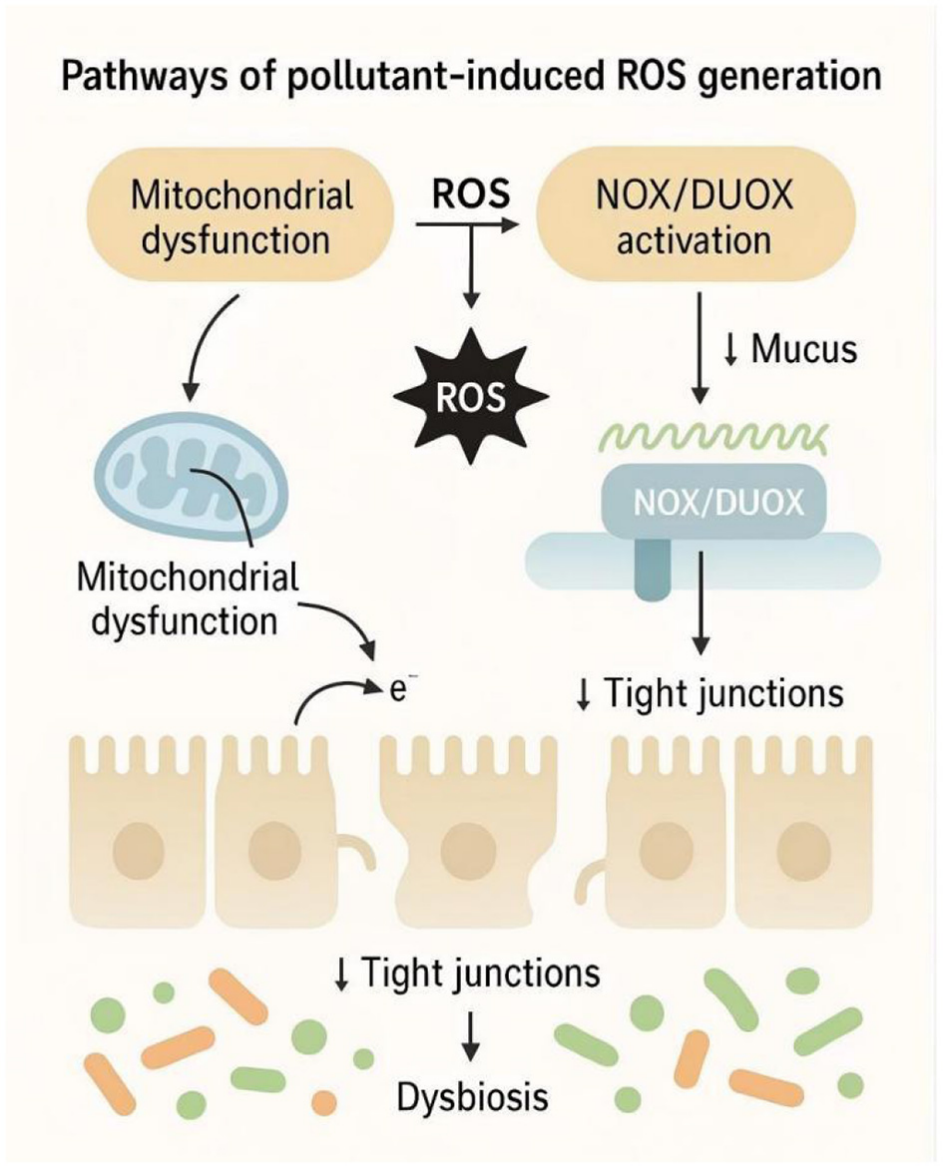
Pathways of pollutant-induced ROS generation affecting gut microbiota. The schematic depicts (1) mitochondrial ROS production after pollutant-induced disruption of the respiratory chain; and (2) NOX/DUOX activation at the epithelial surface. Crosstalk between the two amplifies redox stress, initiating tight-junction injury, mucus depletion, and microecological imbalance that culminate in inflammation.

Oxidative stress is a core mediator linking environmental exposure to gut microbiota dysbiosis and host injury (Samet and Wages, 2018). Pollutants (e.g., heavy metals, micro/nanoplastics, POPs, pesticides, ionizing radiation) elevate reactive oxygen species (ROS) in intestinal epithelial and immune cells along two principal routes: mitochondrial ROS from an impaired electron transport chain, and NADPH oxidase (NOX/DUOX)–derived ROS at the plasma membrane (Huang et al., 2003). Mitochondrial ROS arise when pollutants disturb oxidative phosphorylation, increase electron leakage at complexes I/III, and collapse membrane potential. Then it triggers lipid peroxidation, DNA damage, and apoptosis; NOX1/2/DUOX2 generate superoxide as part of stress and innate defense responses, but sustained activation drives barrier failure and chronic inflammation (Zhang et al., 2014; Hernansanz-Agustín and Enríquez, 2021). Together these pathways initiate redox imbalance that precedes measurable shifts in the gut ecosystem (Figure 5).

Excess ROS weakens epithelial defenses (tight junctions, mucus) and increases paracellular permeability, enabling luminal LPS and other microbe-derived products to access the mucosa and circulation (“leaky gut”; Luchan et al., 2021). This selects for opportunistic and pro-inflammatory taxa while depleting beneficial commensals, a hallmark of dysbiosis. Mechanistically, ROS activates MLCK/actomyosin contraction and NF-κB/MAPK cascades, reducing barrier proteins (e.g., occludin, ZO-1) and sustaining cytokine release, which further perturbs community composition (Fasnacht and Polacek, 2021; Kim et al., 2018).

Besides, microbial signals can themselves modulate epithelial ROS. For example, lactate from symbionts (and traffic through peptidoglycan-recognition pathways) can activate epithelial NOX, increasing ROS and altering stem-cell dynamics and tissue renewal. When excessive, this promotes dysplasia and dysbiosis—illustrating that host–microbe crosstalk can convert local redox signals into ecological change (Lee et al., 2018).

In addition, ROS also regulate metabolic counter-balances. Short-chain fatty acids (SCFAs), notably butyrate, produced by commensal fermentation attenuate oxidative stress, enhance mucin expression, and tighten epithelial permeability partly via G-protein-coupled receptors and the Nrf2/ARE antioxidant axis. Conversely, dysbiosis that lowers SCFA availability diminishes these antioxidant and barrier-protective signals, sensitizing the mucosa to pollutant injury (Mann et al., 2024). Across exposures (e.g., PM/radiation/pesticides), ROS-driven barrier loss and dysbiosis forms the entry point for systemic inflammation and metabolic disturbance via the gut–liver/brain axes. It provides a mechanistic bridge from environmental stressors to whole-body disease phenotypes.

### 3.2 AhR as an integrative hub linking environmental sensing and intestinal immunity

**Figure 6:**
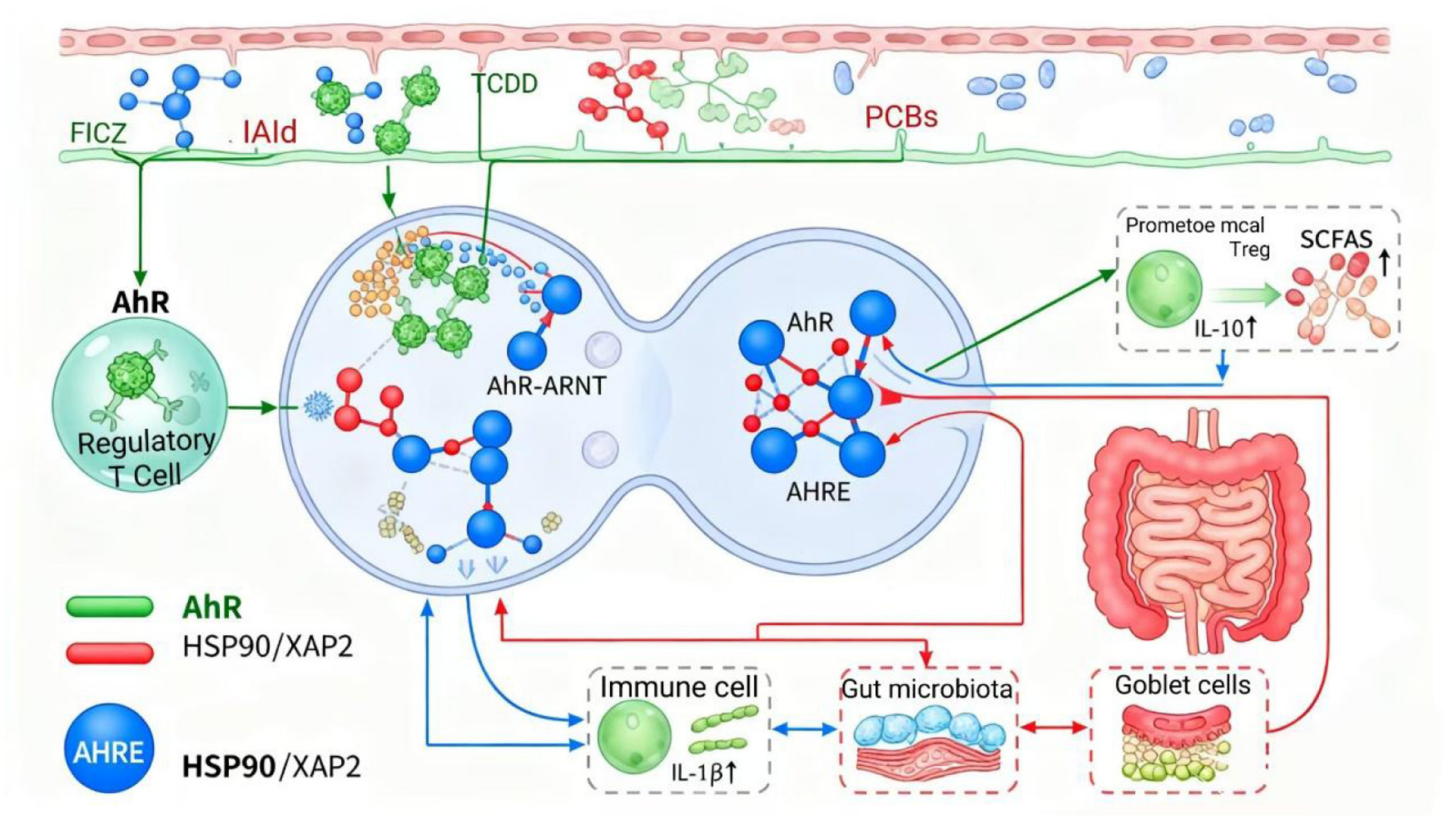
Schematic representation of AhR signaling as a central hub linking environmental pollutants, gut microbiota, and intestinal immunity. Pollutants such as TCDD and PCBs activate AhR in epithelial and immune cells by binding to ligands like FICZ or I3Ald. Upon nuclear translocation, AhR forms a heterodimer with ARNT, binds to AHRE, and alters gene expression. This modulates cytokine production (e.g., IL-10, IL-1β), affects Goblet cell and SCFA dynamics, and alters the microbial composition, collectively influencing gut–immune homeostasis and systemic inflammation.

The aryl hydrocarbon receptor (AhR) signaling pathway serves as a central hub in the toxicological mechanisms of environmental pollutants, bridging xenobiotic metabolism and immune regulation (Narita et al., 2024). In the cytoplasm, AhR typically forms a complex with molecular chaperones such as heat shock protein 90 (HSP90) and X-associated protein 2 (XAP2). Upon ligand binding, either endogenous or exogenous, the receptor complex undergoes conformational change, causing the dissociation of chaperones and facilitating the nuclear translocation of the ligand–AhR complex (Figure 6; Bock, 1994).

Inside the nucleus, AhR dimerizes with the AhR nuclear translocator (ARNT) to form a heterodimer that binds to the aryl hydrocarbon response element (AHRE) on target genes, thereby regulating their transcription. This process affects multiple physiological functions, including xenobiotic metabolism, immune modulation, and intestinal barrier maintenance.

Exogenous pollutants such as dioxins, PCBs, and polycyclic aromatic hydrocarbons (PAHs) act as potent AhR ligands. Persistent activation of AhR by these toxicants leads to the up-regulation of xenobiotic-metabolizing enzymes (CYP1A1, CYP1B1, ALDH3A1) and pro-inflammatory cytokines (IL-6, TNF-α, IL-1β), causing oxidative stress and chronic intestinal inflammation. In contrast, endogenous and microbiota-derived ligands, for example, tryptophan metabolites such as indole-3-aldehyde and indole-3-acetic acid, activate AhR in a transient, physiological manner, promoting IL-22 secretion and epithelial repair to sustain mucosal homeostasis (Chen C. et al., 2024).

Exogenous toxic ligands (e.g., dioxins, PCBs) induce persistent AhR activation, stimulating the expression of CYP1A1, CYP1B1, and inflammatory cytokines (IL-6, TNF-α), which enhance oxidative stress and disturb mucosal balance. In contrast, endogenous or microbial metabolites (e.g., indole derivatives) elicit moderate AhR activation that facilitates IL-22-mediated tissue repair and immunological tolerance. The figure highlights AhR's bidirectional regulatory role and its crosstalk with oxidative and inflammatory pathways in maintaining intestinal homeostasis. Furthermore, AhR signaling is tightly interconnected with redox and immune pathways. Activation of AhR can influence NF-κB and NLRP3 inflammasome activity, forming a feedback network that integrates oxidative stress, inflammation, and microbial signals (Groschwitz and Hogan, 2009). Persistent AhR overstimulation by pollutants disrupts this balance, shifting the intestinal microenvironment from homeostasis toward chronic inflammation and dysbiosis.

### 3.3 Disruption of intestinal barrier integrity

**Figure 7:**
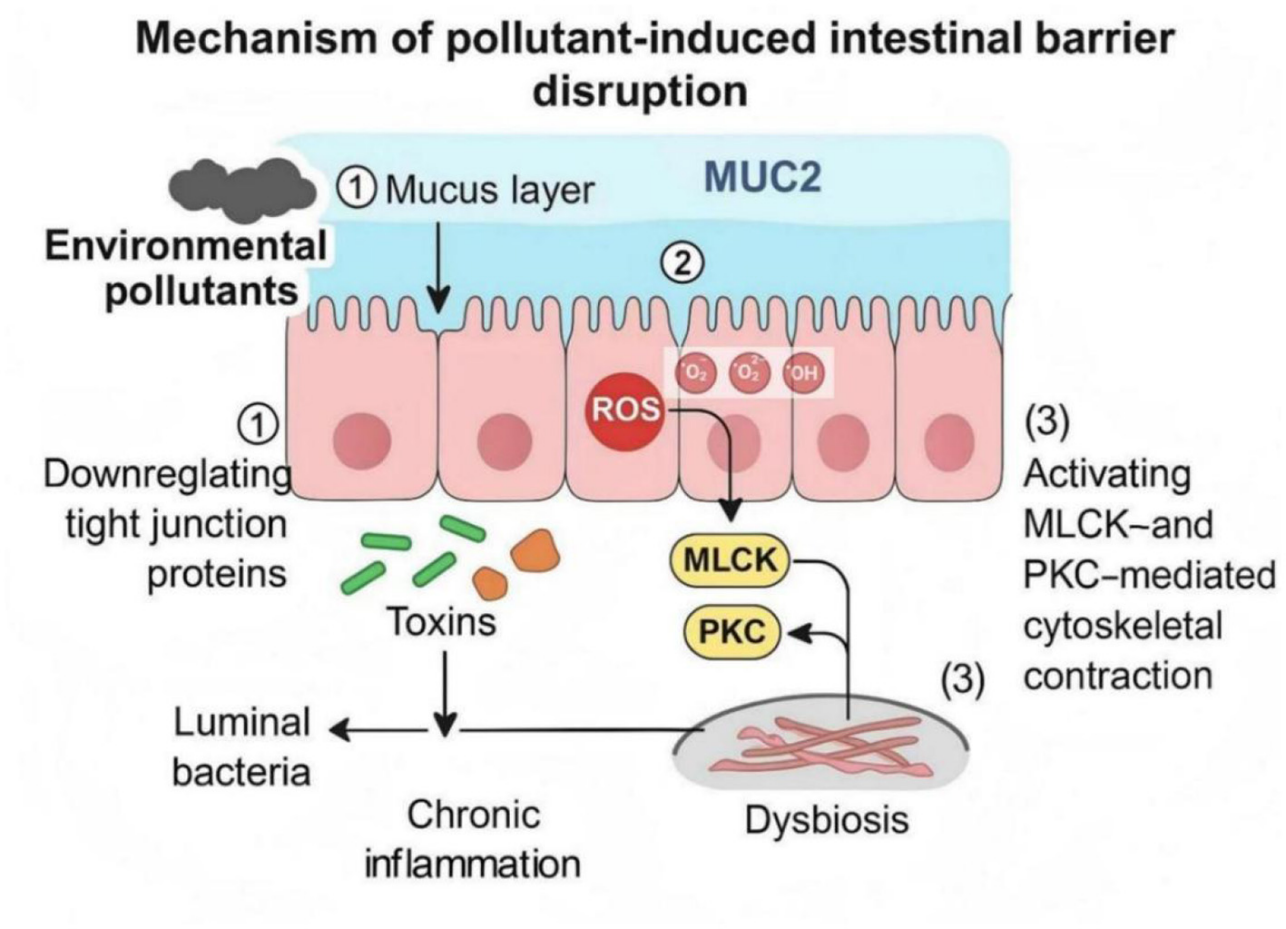
Mechanisms of pollutant-induced intestinal barrier disruption. Environmental pollutants damage the intestinal barrier by **(1)** reducing MUC2 secretion and mucus layer thickness; **(2)** downregulating tight junction proteins such as occludin and ZO-1 via ROS-dependent signaling; and **(3)** activating MLCK- and PKC-mediated cytoskeletal contraction. These effects increase epithelial permeability, facilitating luminal bacterial and toxin translocation and promoting chronic inflammation and dysbiosis.

The intestinal barrier represents the first line of defense between the host and the external environment. It is composed of the mucus layer, epithelial tight junctions, and immune components, all of which work synergistically to maintain intestinal homeostasis (Groschwitz and Hogan, 2009). Environmental pollutants can compromise this complex structure through oxidative stress, inflammatory cytokine overproduction, and direct interference with epithelial proteins, ultimately increasing intestinal permeability and triggering microbial dysbiosis (Figure 7; Song et al., 2023).

The mucus layer forms a physical and biochemical barrier that prevents pathogens and toxins from directly contacting epithelial cells. Pollutants such as bisphenol A (BPA) inhibit the secretion of mucin 2 (MUC2) in goblet cells by inducing mitochondrial dysfunction and oxidative stress, leading to a thinner mucus layer and increased vulnerability to bacterial invasion (Zhao et al., 2019). The transcriptional regulation of MUC2 involves factors such as p53, and impairment of its expression weakens mucosal protection (Ookawa et al., 2002). Clinical and experimental evidence suggests that structural weakening of the mucus barrier is an early pathological event in ulcerative colitis (UC), which facilitates microbial translocation and inflammation (van der Post et al., 2019).

In addition to mucus reduction, pollutants also degrade tight junction proteins, disrupting intercellular integrity. Exposure to particulate matter (PM_2.5_) and heavy metals such as cadmium (Cd) or lead (Pb) activates oxidative stress–dependent enzymes that degrade tight junction proteins including occludin, claudin, and ZO-1, resulting in increased epithelial permeability (Wang et al., 2012). The activation of myosin light chain kinase (MLCK) and protein kinase C (PKC) induces cytoskeletal contraction, further expanding paracellular gaps and enhancing intestinal permeability (He et al., 2020).

Persistent barrier dysfunction promotes the leakage of lipopolysaccharides (LPS) and other microbial products into systemic circulation, where they bind to Toll-like receptor 4 (TLR4) on immune cells, initiating NF-κB–mediated inflammatory cascades (Olivera et al., 2010). This “leaky gut” condition contributes to endotoxemia, metabolic disturbance, and immune dysregulation, creating a vicious cycle between inflammation and microbial imbalance.

Therefore, the intestinal barrier serves as both a target and a mediator of pollutant-induced toxicity. Its structural and functional disruption not only disturbs host-microbe interactions but also accelerates systemic inflammation, forming a crucial mechanistic link between environmental exposure, oxidative injury, and gut microbiota dysbiosis.

### 3.4 Amplification and persistence of immune-inflammatory responses

**Figure 8:**
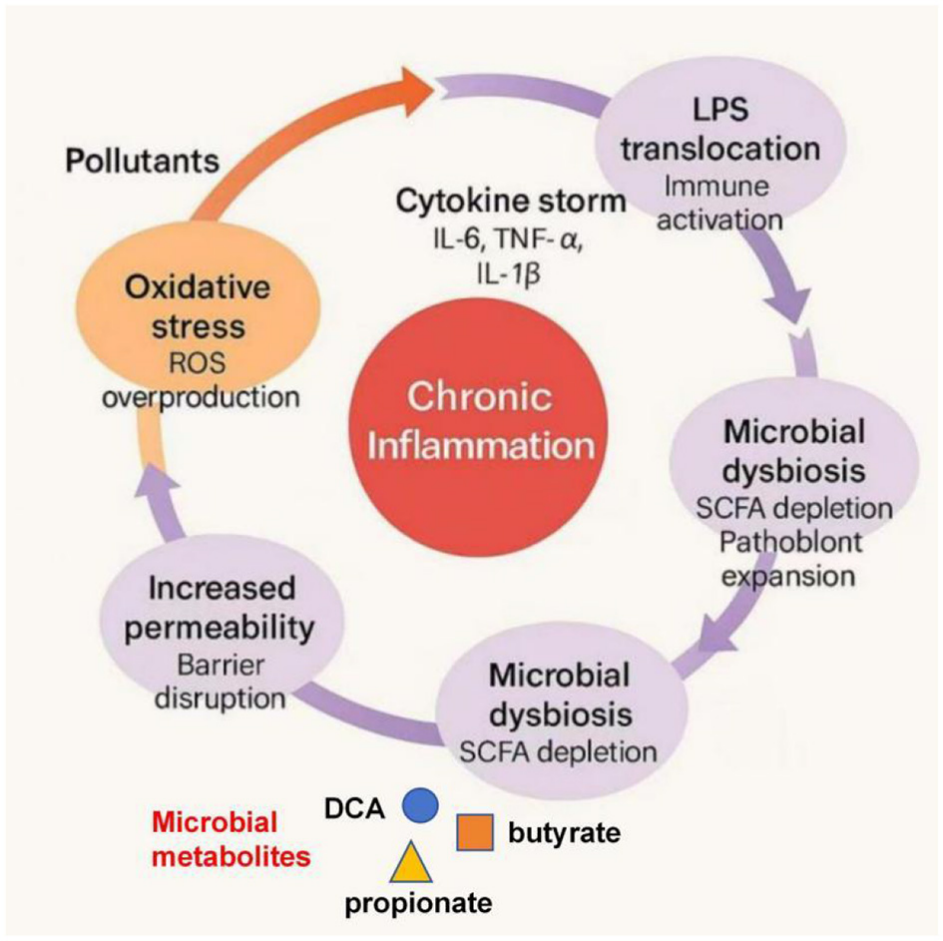
Positive feedback loops driving pollutant-induced chronic inflammation. Pollutant-induced oxidative stress (via ROS overproduction) compromises epithelial barrier integrity, increasing intestinal permeability. This facilitates LPS translocation and immune activation, driving microbial dysbiosis (e.g., SCFA depletion) and sustained cytokine release (IL-6, TNF-α, IL-1β), thereby perpetuating chronic inflammation.

Following oxidative stress and barrier disruption, environmental pollutants initiate a cascade of immune activation that can progress from acute mucosal inflammation to chronic systemic dysregulation (Samak et al., 2014). Persistent exposure sustains cytokine production, recruits immune cells, and alters the intestinal microbiota, forming a self-reinforcing cycle of inflammation and oxidative injury (Figure 8).

At the early stage, pattern recognition receptors (PRRs), notably TLR4, detect bacterial LPS or pollutant-associated molecular patterns. This triggers downstream MyD88–NF-κB and MAPK pathways, leading to the production of IL-6, TNF-α, and IL-1β (Guo et al., 2015). While these mediators are essential for pathogen clearance, their persistent expression transforms transient immune defense into chronic inflammation. Pollutants such as heavy metals and polycyclic aromatic hydrocarbons (PAHs) can prolong this activation by upregulating NLRP3 inflammasome components, resulting in excessive secretion of IL-1β and IL-18 (Chen et al., 2002).

Macrophages and dendritic cells play pivotal roles in amplifying pollutant-induced inflammation. Upon ROS stimulation, macrophages polarize toward the M1 phenotype, producing proinflammatory mediators, while suppressing anti-inflammatory M2 differentiation (Maes et al., 2008). This skewed polarization enhances tissue damage and sustains cytokine production. Moreover, pollutants alter the balance of T helper (Th) cell subsets: exposure to dioxins or BPA increases Th17 differentiation and IL-17A release while suppressing Treg function and IL-10 signaling, weakening the mucosal anti-inflammatory network (Alexander and Rietschel, 2001).

Pollutants and microbial LPS activate TLR4/NF-κB and NLRP3 inflammasome pathways, leading to sustained IL-6, TNF-α, and IL-1β production. Reactive oxygen species generated by mitochondria and NADPH oxidases further enhance these signals. Simultaneously, dysbiosis reduces beneficial metabolites such as butyrate and indole derivatives, impairing Treg differentiation and IL-10 synthesis (Zamyatina and Heine, 2021). The resulting imbalance between pro- and anti-inflammatory responses creates a persistent inflammatory microenvironment that reinforces oxidative stress and barrier injury.

Microbial dysbiosis exacerbates this inflammatory persistence. Pollutant-induced loss of SCFA-producing bacteria diminishes butyrate-mediated inhibition of histone deacetylases (HDACs), which normally suppress NF-κB–driven inflammation (Küper et al., 2012). Similarly, the depletion of tryptophan-metabolizing commensals reduces the generation of AhR ligands (e.g., indole-3-aldehyde), weakening IL-22–mediated mucosal repair and further amplifying the inflammatory loop (Andersen, 2016). These microbiota-driven alterations transform transient immune responses into sustained pathology.

At the systemic level, translocated bacterial endotoxins and inflammatory cytokines enter circulation, activating hepatic Kupffer cells and microglia through gut–liver and gut–brain axes (Lin et al., 2025). This mechanism contributes to extra-intestinal complications, including non-alcoholic fatty liver disease (NAFLD), neuroinflammation, and metabolic syndrome, emphasizing that intestinal immune activation is a key driver of systemic pollutant toxicity.

In conclusion, pollutant-induced immune responses evolve from acute epithelial inflammation to chronic systemic activation through a network of positive feedback loops involving ROS, cytokine signaling, and microbiota dysbiosis. Understanding these reinforcing mechanisms provides a foundation for developing anti-inflammatory and microbiota-targeted strategies to mitigate chronic pollutant-induced disorders.

## 4 Gut microbiota in health and disease

### 4.1 Metabolic disorders

The gut microbiota plays a central role in host energy balance and metabolic regulation by participating in SCFA production, bile acid transformation, and glucose–lipid metabolism (Winter et al., 2013). Environmental pollutants disrupt these microbial pathways, leading to altered metabolite profiles and metabolic dysfunctions such as obesity, insulin resistance, and non-alcoholic fatty liver disease.

#### 4.1.1 Microbial dysbiosis and metabolic imbalance

Exposure to pollutants such as PM_2.5_, BPA, and heavy metals (Cd, Pb) markedly alters microbial community composition, reducing the abundance of beneficial SCFA-producing genera (*Faecalibacterium, Roseburia, Akkermansia*) while expanding opportunistic taxa such as *Desulfovibrio* and *Enterobacteriaceae* (Wu et al., 2022; Klemenak et al., 2015; Moreira de Gouveia et al., 2024). These compositional changes result in lower levels of butyrate and propionate, key metabolites that regulate intestinal epithelial energy supply and systemic glucose homeostasis through the GPR41/43–AMPK axis (Zhang et al., 2022). Butyrate depletion weakens epithelial integrity, promotes low-grade endotoxemia, and triggers chronic inflammation—recognized hallmarks of metabolic disease progression.

#### 4.1.2 Pollutant–microbiota–host metabolic interactions

Environmental toxicants interfere with microbial metabolism in multiple ways: (1) disruption of bile acid metabolism: pollutants alter bacterial bile salt hydrolase (BSH) activity, affecting the ratio of primary to secondary bile acids and impairing signaling through farnesoid X receptor (FXR) and TGR5, both critical for lipid and glucose regulation (Liu et al., 2025); (2) induction of endotoxemia and insulin resistance: increased intestinal permeability following pollutant exposure allows translocation of LPS into circulation, activating TLR4–NF-κB signaling in adipose and hepatic tissues. This inflammatory activation reduces insulin receptor sensitivity and accelerates metabolic syndrome (Kim et al., 2012); and (3) oxidative and mitochondrial stress: heavy metals and POPs impair mitochondrial β-oxidation and ATP production, thereby altering energy metabolism and promoting lipid accumulation (Zhan et al., 2022).

#### 4.1.3 Microbiota-mediated metabolic memory and disease susceptibility

Accumulating evidence suggests that pollutant-induced dysbiosis may reprogram host metabolic responses over the long term, a phenomenon often referred to as microbiota-mediated metabolic memory. Current support for this concept is derived largely from animal models and mechanistic studies, with limited direct evidence in humans. For example, perinatal exposure to bisphenol A (BPA) or polybrominated diphenyl ethers (PBDEs) has been shown in rodent models to alter early-life microbial colonization and to associate with epigenetic modifications of lipid metabolism–related genes, including PPARγ and SREBP-1c, thereby predisposing offspring to obesity and glucose intolerance (López-Moreno et al., 2025). Similarly, chronic PM_2.5_ exposure in experimental systems shifts the intestinal microbiome toward a pro-obesogenic profile, characterized by elevated branched-chain amino acids (BCAAs) and reduced short-chain fatty acids (SCFAs), which correlates with aggravated insulin resistance (Salehi et al., 2010).

**Figure 9:**
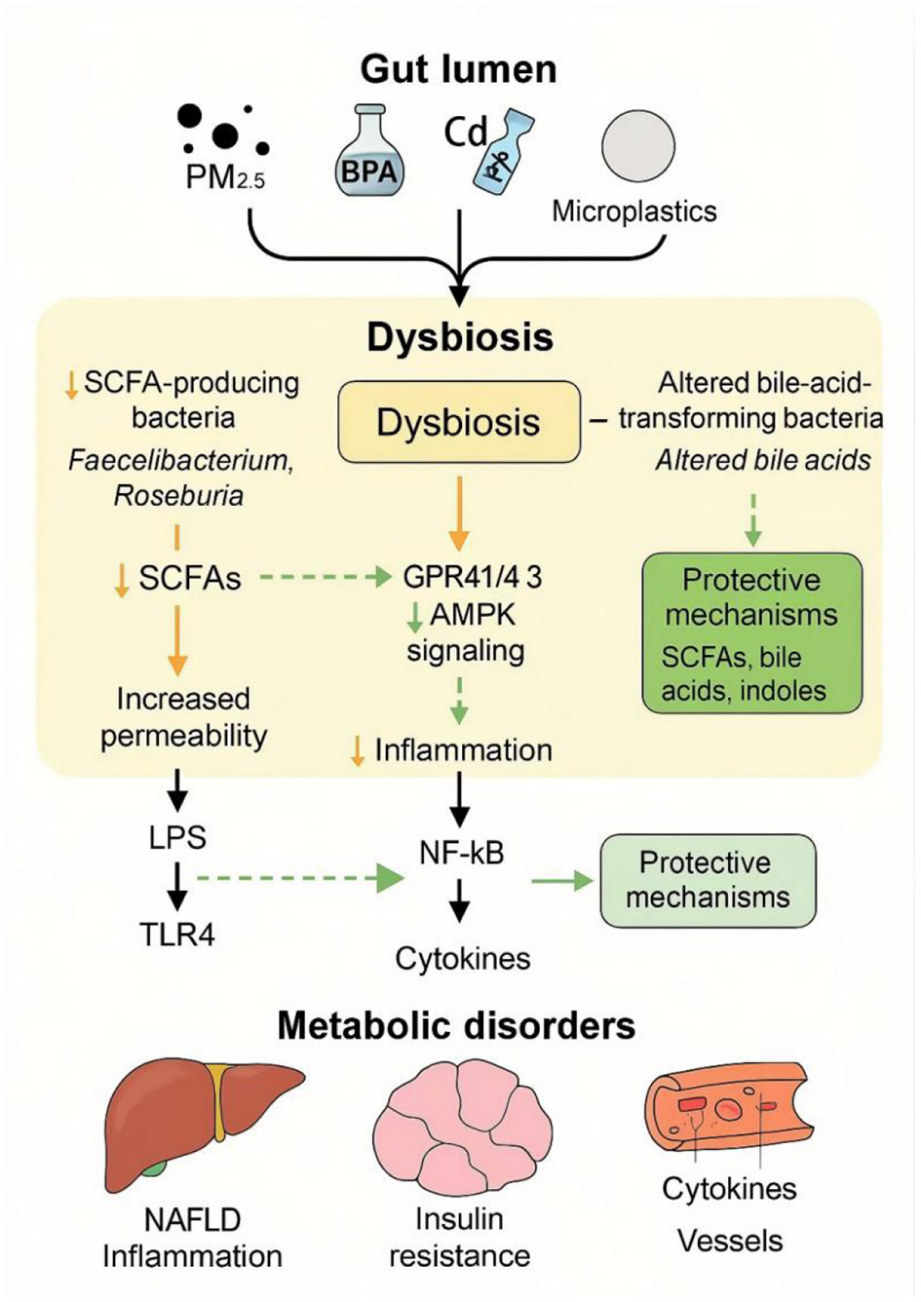
Gut microbiota-mediated pathways linking pollutant exposure to metabolic disorders. Exposure to environmental pollutants such as PM_2.5_, bisphenol A (BPA), cadmium (Cd), and microplastics disrupts the intestinal microbial balance (dysbiosis) by reducing SCFA-producing bacteria (e.g., *Faecalibacterium, Roseburia*) and altering bile acid-transforming bacteria. These changes lead to decreased SCFAs, increased intestinal permeability, and activation of TLR4 signaling via LPS translocation. Concurrently, reduced SCFA-GPR41/43-AMPK signaling and enhanced NF-κB–mediated inflammation contribute to cytokine overproduction. These perturbations promote systemic inflammation and metabolic disorders including non-alcoholic fatty liver disease (NAFLD), insulin resistance, and vascular inflammation. Protective mechanisms, such as SCFAs, bile acids, and indoles, may partially counteract this pathogenic cascade.

Collectively, these findings support the hypothesis that the gut microbiota can function as a metabolic and epigenetic interface translating environmental exposures into altered disease susceptibility. Mechanistically, environmental pollutants disrupt microbial communities and key metabolic circuits by reducing SCFA synthesis, altering bile acid transformation, and increasing lipopolysaccharide (LPS) translocation. These microbial perturbations may impair FXR/TGR5 and GPR41/43 signaling, thereby promoting insulin resistance, lipid accumulation, and chronic low-grade inflammation, ultimately contributing to metabolic disorders such as obesity, type 2 diabetes, and non-alcoholic fatty liver disease (NAFLD; Figure 9).

However, it is important to note that although pathways such as SCFA–GPR41/43 and AhR–IL-22 signaling have been repeatedly implicated in pollutant-associated metabolic dysregulation, the majority of mechanistic evidence originates from animal or *in vitro* studies. For instance, reduced butyrate levels are consistently associated with impaired barrier function and insulin resistance, yet direct causal relationships in human populations remain limited (Perl et al., 2025). Likewise, the immunometabolic roles of microbial tryptophan-derived AhR ligands are well established in murine models, but their contribution to long-term metabolic programming in humans has not been definitively demonstrated (Duan et al., 2025). In addition, substantial inter-individual variability including genetic background, dietary patterns, and baseline microbiota composition may modulate these pathways and influence susceptibility in complex and context-dependent manners (Chen L. et al., 2022). Therefore, while microbiota-mediated metabolic memory represents a compelling conceptual framework, further longitudinal human studies integrating multi-omics profiling are required to establish causality, define dose–response relationships, and determine the persistence of microbiota-driven metabolic alterations following pollutant exposure.

### 4.2 Immune and inflammatory disorders

The intestinal microbiota serves as a crucial regulator of mucosal and systemic immunity by shaping immune cell maturation, cytokine secretion, and tolerance mechanisms. Environmental pollutants disturb this finely tuned microbiota–immune axis, leading to immune hyperactivation, chronic inflammation, and increased susceptibility to immune-related diseases (Shen et al., 2025).

#### 4.2.1 Pollutant-induced immune imbalance through gut dysbiosis

Pollutant exposure profoundly alters microbial composition and metabolite signaling. Loss of *Lactobacillus, Bifidobacterium*, and *Akkermansia muciniphila*—key taxa involved in immunomodulation—reduces the production of short-chain fatty acids (SCFAs) and indole derivatives, both known to promote regulatory T cell (Treg) differentiation and IL-10 secretion (Pingitore et al., 2019). This metabolic deprivation weakens mucosal immune tolerance and skews immune balance toward proinflammatory Th1/Th17 responses.

**Figure 10:**
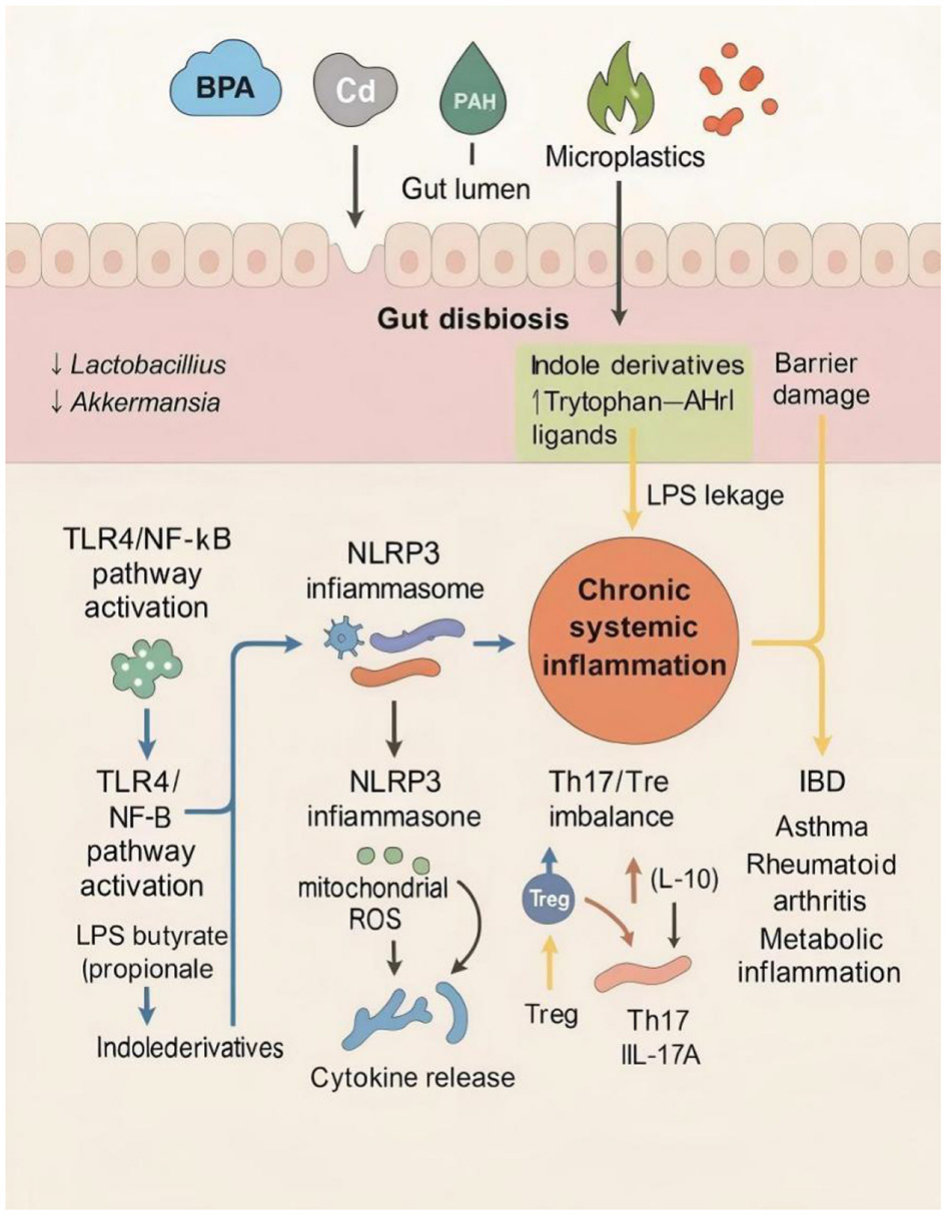
Gut microbiota-mediated pathways linking pollutant exposure to immune and inflammatory dysregulation. Environmental pollutants such as BPA, cadmium (Cd), polycyclic aromatic hydrocarbons (PAHs), and microplastics disrupt gut microbial homeostasis, leading to dysbiosis characterized by decreased beneficial genera (e.g., *Lactobacillus, Akkermansia*) and altered microbial metabolites (e.g., increased indole derivatives and tryptophan–AhR ligands). This imbalance impairs intestinal barrier function and promotes lipopolysaccharide (LPS) leakage. LPS and microbial metabolites activate the TLR4/NF-κB pathway and NLRP3 inflammasome, resulting in mitochondrial ROS production and cytokine release. These processes collectively contribute to chronic systemic inflammation and a Th17/Treg imbalance, ultimately increasing the risk of immune-mediated and metabolic diseases, including inflammatory bowel disease (IBD), asthma, rheumatoid arthritis, and metabolic inflammation.

In parallel, pollutants such as bisphenol A (BPA), cadmium, and PAHs activate pattern recognition receptors (PRRs), such as TLR on intestinal epithelial and immune cells, stimulating NF-κB and MAPK pathways. The resulting cytokine surge (IL-6, TNF-α, IL-1β) promotes local inflammation, recruits neutrophils and macrophages, and compromises barrier function (Song et al., 2025; Xi et al., 2019). Persistent activation of NLRP3 inflammasome further amplifies this inflammatory cascade, leading to chronic tissue injury (Figure 10; Paik et al., 2025).

Environmental pollutants alter gut microbial composition, decreasing SCFA and indole metabolite levels that normally sustain Treg/IL-10 anti-inflammatory signaling. Concurrently, pollutant-activated TLR4/NF-κB and NLRP3 pathways elevate proinflammatory cytokines (IL-6, TNF-α, IL-17), disrupt the Th17/Treg balance, and impair epithelial integrity. These synergistic effects promote chronic intestinal inflammation and heighten systemic immune reactivity, predisposing to immune-mediated diseases.

#### 4.2.2 Mechanistic insights: the Th17/Treg and AhR axes

A hallmark of pollutant-induced immune dysfunction is the disruption of the Th17/Treg balance. BPA and dioxins enhance Th17 differentiation via STAT3 and RORγt activation while suppressing Foxp3^+^ Treg expansion, promoting sustained inflammation (Grover et al., 2021). Meanwhile, dysbiosis-driven depletion of tryptophan-derived AhR ligands (e.g., indole-3-aldehyde, FICZ) impairs IL-22 production, compromising epithelial defense and mucosal repair (Bahman et al., 2024). These findings underscore AhR's dual role as both a pollutant sensor and an immunoregulatory gatekeeper.

#### 4.2.3 From intestinal inflammation to systemic immune disorders

Chronic pollutant exposure results in translocation of microbial products such as lipopolysaccharides (LPS) and peptidoglycans into circulation, eliciting systemic immune activation. Epidemiological studies associate prolonged exposure to PM_2.5_, heavy metals, and microplastics with increased incidence of inflammatory bowel disease (IBD), asthma, rheumatoid arthritis, and multiple sclerosis (Li F. R. et al., 2022; Liu et al., 2022; Sharif and Amital, 2014). Mechanistically, dysbiosis-induced cytokine overflow and ROS generation contribute to the systemic spread of inflammation through the gut–liver and gut–brain axes, forming a molecular bridge between environmental exposure and autoimmune pathogenesis.

### 4.3 Neurological disorders

The gut microbiota exerts profound influence on the central nervous system (CNS) through the microbiota–gut–brain axis, a bidirectional communication network involving neural, endocrine, and immune pathways (Smith and Vale, 2006). Environmental pollutants disrupt this delicate axis by altering microbial composition and metabolic output, thereby modulating neurotransmission, neuroinflammation, and cognitive function (Heidari and Lawrence, 2024).

#### 4.3.1 Microbiota dysbiosis and neuroimmune activation

**Figure 11:**
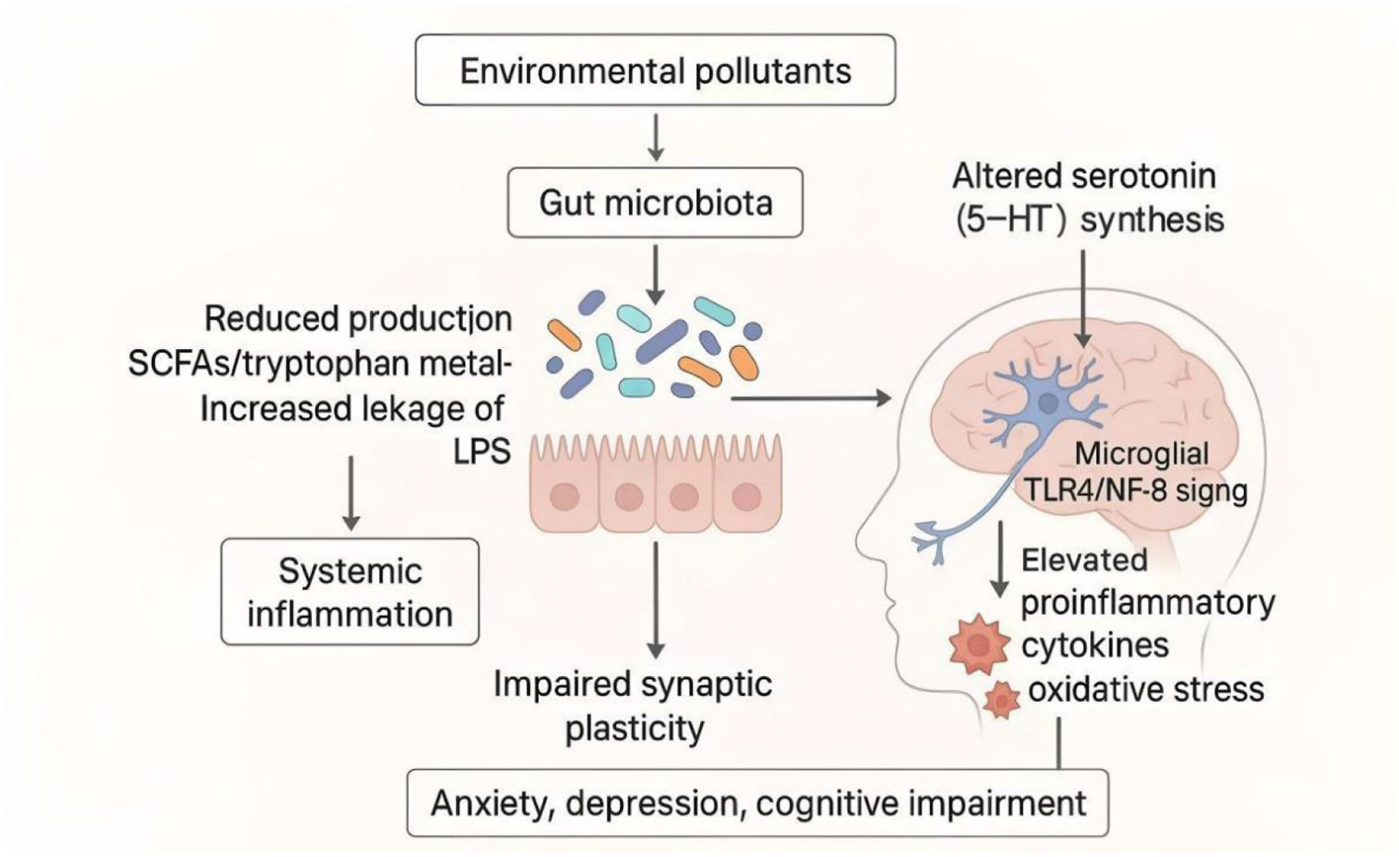
Gut microbiota-mediated pathways linking pollutant exposure to neurotoxicity. Environmental pollutants disrupt the gut microbiota, reducing SCFA and tryptophan metabolite production while increasing LPS leakage. These changes activate systemic inflammation and microglial TLR4/NF-κB signaling, leading to elevated proinflammatory cytokines and oxidative stress in the brain. Altered serotonin (5-HT) synthesis, impaired synaptic plasticity, and neuroinflammation collectively contribute to anxiety, depression, and cognitive impairment.

Pollutant-induced gut dysbiosis reduces the abundance of beneficial taxa such as *Lactobacillus* and *Bifidobacterium*, which are essential for producing neuroactive metabolites including SCFAs and tryptophan derivatives (Kelly et al., 2017). Importantly, these bacteria also synthesize γ-aminobutyric acid (GABA) and serotonin precursors: for example, *Lactobacillus rhamnosus* and *Bifidobacterium dentium* produce GABA via glutamate decarboxylation, while *Enterococcus* and *Streptococcus* species contribute to 5-hydroxytryptophan (5-HTP) production from tryptophan (Kelly et al., 2017). SCFAs, particularly butyrate and propionate, modulate microglial maturation and anti-inflammatory function via GPR41/43–HDAC inhibition pathways. Their depletion leads to exaggerated microglial activation, elevated proinflammatory cytokines (IL-6, IL-1β, TNF-α), and impaired synaptic plasticity (Figure 11; Sun et al., 2025).

Pollutants such as PM_2.5_, BPA, and heavy metals enhance intestinal permeability (“leaky gut”), facilitating systemic translocation of LPS and other microbial products that trigger neuroinflammation through TLR4–NF-κB signaling in brain-resident microglia (Sangkham et al., 2024; Bolan et al., 2021). This neuroimmune activation is accompanied by oxidative stress and mitochondrial dysfunction, key contributors to neuronal injury and cognitive decline.

#### 4.3.2 Tryptophan metabolism and neurotransmitter imbalance

Tryptophan metabolism is a key microbial–neuronal interface. Dysbiosis reduces conversion of tryptophan to indole-3-aldehyde and indole-3-propionic acid, weakening AhR–IL-22–mediated neuroprotection, while diverting tryptophan toward the kynurenine pathway, which generates neurotoxic metabolites such as quinolinic acid (Hou et al., 2023). Furthermore, gut bacteria such as *Escherichia coli, Clostridium sporogenes*, and *Bacteroides fragilis* play central roles in regulating host serotonin levels by modulating peripheral tryptophan metabolism. Dysbiosis-associated reductions in these taxa may impair 5-HT availability, contributing to emotional and behavioral disturbances including anxiety and depression (Zundel et al., 2022). These effects are further amplified by compromised vagal signaling and disrupted hypothalamic–pituitary–adrenal (HPA) axis feedback.

#### 4.3.3 Evidence linking pollutant exposure to neurobehavioral outcomes

Both animal and epidemiological studies demonstrate pollutant-related neurological consequences mediated by gut dysbiosis: (1) chronic exposure to diesel exhaust particles alters microbial diversity, enhances microglial activation, and induces memory impairment and depressive-like behaviors in mice (Phillippi et al., 2022); (2) BPA and phthalates interfere with microbiota-driven serotonin metabolism, correlating with increased risk of autism spectrum disorder (ASD) and attention-deficit/hyperactivity disorder (ADHD) in children (Martínez-Ibarra et al., 2021); and (3) lead and cadmium exposure disrupt gut–brain communication by damaging enteric neurons and modifying microbial neurochemical signaling, contributing to cognitive and motor deficits (Porru et al., 2024).

### 4.4 Carcinogenesis and cancer susceptibility

Chronic exposure to environmental pollutants significantly increases the risk of carcinogenesis, in part through gut microbiota–mediated mechanisms that shape inflammatory, metabolic, and genotoxic microenvironments (Wang et al., 2020b). The gut microbiota acts as both a metabolic converter and an inflammatory amplifier, transforming xenobiotics into carcinogenic metabolites while sustaining a pro-tumorigenic milieu in the intestine and distal organs.

#### 4.4.1 Microbial metabolism and carcinogenic intermediates

**Figure 12:**
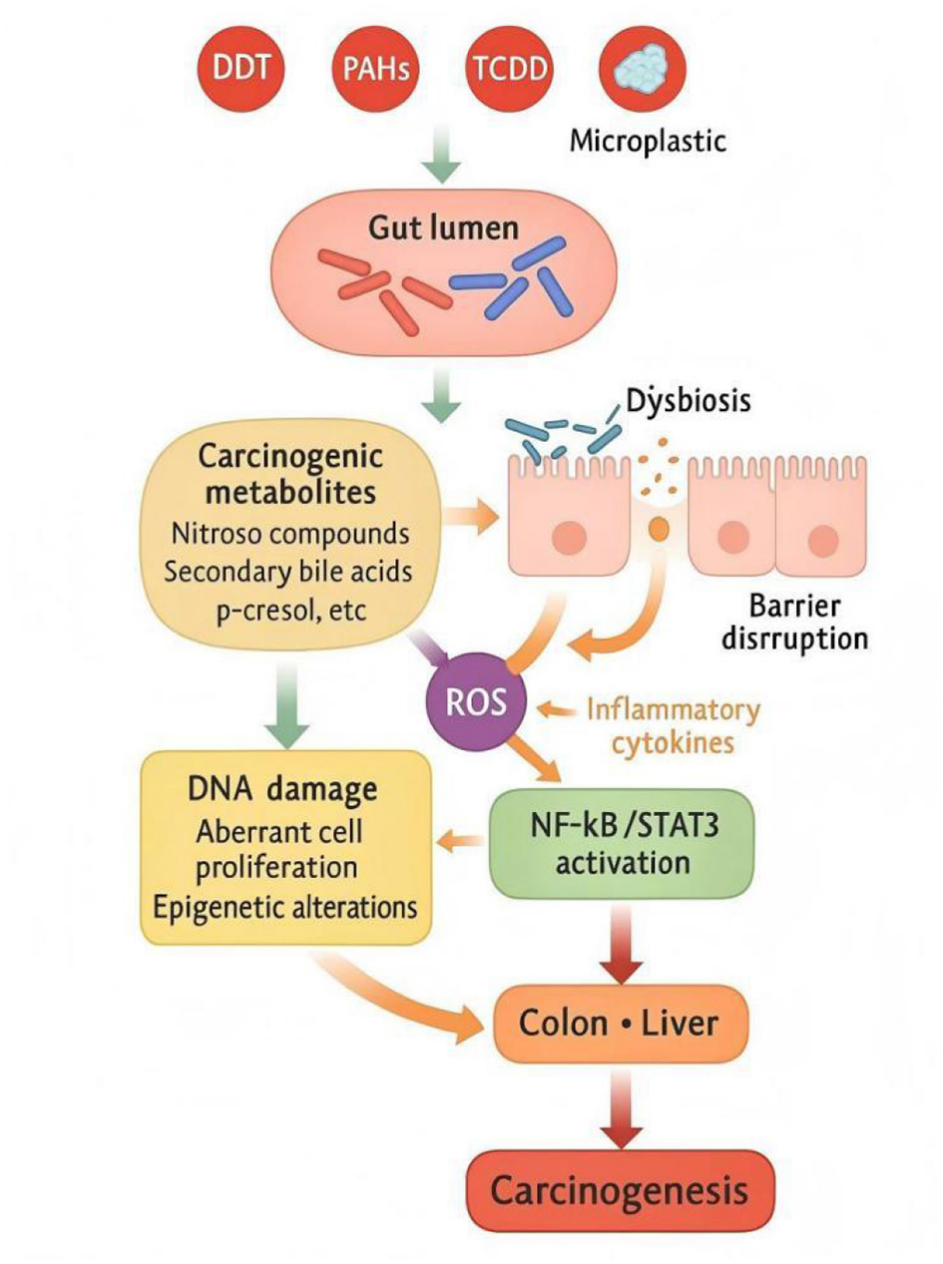
Gut microbiota-mediated pathways linking environmental pollutants to carcinogenesis. Environmental pollutants are metabolized by dysbiotic microbiota into carcinogenic intermediates (nitroso compounds, secondary bile acids). These metabolites induce oxidative stress and chronic inflammation through NF-κB/STAT3 activation, leading to DNA damage, aberrant cell proliferation, and epigenetic reprogramming. The sustained inflammatory–oxidative microenvironment enhances susceptibility to colorectal and hepatocellular carcinoma.

Environmental pollutants such as PAHs, PCBs, and heavy metals (As, Cd) undergo microbial transformation in the gut, producing reactive intermediates that damage host DNA (Shimada and Fujii-Kuriyama, 2004; Rezazadegan et al., 2025). Dysbiotic microbiota exhibit increased nitroreductase, β-glucuronidase, and azoreductase activities, generating nitroso compounds and aromatic amines, both established mutagens in colorectal carcinogenesis (Boddu et al., 2021). Concurrently, disruption of bile acid metabolism elevates secondary bile acids such as deoxycholic acid (DCA) and lithocholic acid (LCA), which induce oxidative stress, activate NF-κB and STAT3, and promote cell proliferation and DNA damage (Figure 12; Shi et al., 2023).

#### 4.4.2 Chronic inflammation, oxidative stress, and DNA damage

Pollutant-induced dysbiosis perpetuates low-grade inflammation and oxidative stress, two hallmarks of cancer initiation (Rio et al., 2024). Persistent activation of immune pathways (e.g., TLR4/NF-κB, NLRP3 inflammasome) increases IL-6 and TNF-α, stimulating epithelial proliferation and angiogenesis. Meanwhile, ROS generated by both pollutants and inflammatory cells cause DNA strand breaks, lipid peroxidation, and the formation of mutagenic adducts such as 8-hydroxy2′-deoxyguanosine (8-OHdG; Yu et al., 2024). These genotoxic events accumulate over time, facilitating oncogenic mutations and chromosomal instability.

#### 4.4.3 Epigenetic and metabolic reprogramming

Beyond direct mutagenesis, environmental pollutants induce epigenetic alterations mediated by microbiota-derived metabolites. Aberrant DNA methylation of tumor suppressor genes (p53, APC) and histone acetylation imbalance have been observed following exposure to Cd or PCBs, correlating with shifts in microbial SCFA production (Geissler et al., 2024). Moreover, dysregulated tryptophan–AhR signaling alters intestinal stem cell proliferation, linking microbial metabolism to tumor promotion (Zhu and Van den Eynde, 2024). These findings suggest that the gut microbiota not only mediates pollutant metabolism but also reprograms host gene expression, contributing to long-term cancer susceptibility.

## 5 Strategies and perspectives

### 5.1 Mechanistic insights and current knowledge gaps

Over the past decade, substantial evidence has demonstrated that environmental pollutants exert systemic toxicity through gut microbiota disruption, reshaping host metabolism, immunity, and disease susceptibility. However, despite the emerging conceptual clarity, several critical mechanistic and methodological gaps remain unresolved. This may partly reflect the historical dominance of organ-centric toxicology models that inadequately capture microbiota-mediated systemic effects.

First, the causal hierarchy between pollutant exposure, microbial dysbiosis, and host pathology is not fully delineated. Most studies rely on correlation-based microbiome analyses without disentangling direct toxic effects from microbiota-mediated mechanisms. Integrative approaches, such as germ-free and gnotobiotic animal models, microbiota transplantation, and multi-omics integration (metagenomics, metabolomics, and epigenomics) are urgently needed to establish causality and dose–response relationships.

Second, real-world exposure scenarios typically involve chronic, low-dose, and mixed pollutants (e.g., PM_2.5_, BPA, Cd, microplastics), yet most experimental designs focus on single high-dose pollutants. These simplified models overlook the synergistic or antagonistic interactions among pollutants, as well as host factors such as age, sex, diet, and genetics that shape microbiota resilience. Notably, most existing studies focus on single pollutants, whereas real-world exposures often involve mixtures. Co-exposure to agents like heavy metals and POPs can have additive or even synergistic effects on gut microbiota, leading to more severe dysbiosis and barrier disruption. Furthermore, host factors such as diet, genetics, age, and sex influence susceptibility to microbial alterations. For instance, high-fiber diets may mitigate pollutant-induced SCFA depletion, while aging is linked to reduced microbial resilience. Genetic variations in immune or detoxification pathways may also shape individual responses. Future studies should therefore adopt multi-pollutant models and account for inter-individual variability to better reflect real-world conditions. Advanced computational modeling and exposome–microbiome correlation networks may help capture the complexity of environmental exposure biology.

Third, while key mechanistic pathways including oxidative stress, inflammatory signaling, intestinal barrier damage, and AhR modulation have been identified, their temporal dynamics and cross-tissue effects remain poorly understood. For instance, how intestinal oxidative stress propagates to the brain or liver through microbiota-derived metabolites (e.g., SCFAs, bile acids, indoles) requires more precise spatiotemporal mapping using single-cell and imaging-based approaches.

Finally, standardization issues persist across studies. Differences in sampling depth, sequencing platforms, bioinformatic pipelines, and animal housing conditions hinder data comparability. Establishing reference microbiomes, standard exposure protocols, and global pollutant–microbiome databases would enhance reproducibility and facilitate meta-analyses across populations.

### 5.2 Microbiota-targeted intervention strategies

Given the central role of the gut microbiota in mediating pollutant toxicity, microbiota-targeted prevention and therapy represent promising strategies for mitigating health impacts. These strategies can be classified into three major categories: microbial modulation, dietary interventions, and personalized microbiome medicine.

#### 5.2.1 Probiotics and synbiotics

Probiotics such as *Lactobacillus plantarum, L. rhamnosus*, and *Bifidobacterium longum* have demonstrated efficacy in mitigating cadmium, BPA, and PM_2.5_ toxicity by restoring microbial balance, enhancing intestinal barrier integrity, and reducing oxidative stress. When combined with prebiotics such as inulin or galacto-oligosaccharides, these synbiotic formulations further improve colonization stability and functional resilience. Future research should emphasize strain-specific functions, such as pollutant-binding capacity, SCFA synthesis, or ROS scavenging activity.

#### 5.2.2 Dietary and functional nutrition approaches

Dietary modulation profoundly influences the microbiome's response to pollutants. Diets rich in fermentable fibers, polyphenols, and omega-3 fatty acids promote beneficial microbial taxa and anti-inflammatory metabolites. For example, resveratrol and curcumin attenuate PCB-induced oxidative injury by activating the Nrf2–Keap1 pathway, while green tea catechins counteract heavy metal toxicity through chelation and microbial detoxification. Designing nutritional countermeasures that harness microbiota-derived metabolites (e.g., butyrate, indole derivatives) may provide sustainable protection against pollutant exposure.

#### 5.2.3 Fecal microbiota transplantation (FMT) and next-generation probiotics

Emerging studies suggest that fecal microbiota transplantation (FMT) can reverse pollutant-induced metabolic and inflammatory disorders by re-establishing microbial homeostasis. Moreover, next-generation probiotics (NGPs) including *A. muciniphila, Faecalibacterium prausnitzii*, and engineered *Lactobacillus* strains offer targeted modulation of intestinal redox and immune environments. Integrating FMT and NGPs with precision diagnostics (e.g., microbiome signatures predictive of exposure response) could enable personalized microbiome therapies.

#### 5.2.4 Environmental and policy implications

From a broader perspective, microbiome research should inform environmental risk assessment and policy. Incorporating microbial endpoints (e.g., microbiota diversity, SCFA levels, functional resilience) into pollutant toxicity evaluation may provide more sensitive biomarkers for ecological and human health monitoring. Policies that integrate pollutant reduction, microbiota-friendly agriculture, and sustainable food systems will be crucial to maintaining microbial and planetary health.

## 6 Conclusions

Looking forward, research on pollutant–microbiota interactions will likely evolve toward systems-level integration and translational application. The convergence of multi-omics, artificial intelligence (AI), and exposomics will enable precise mapping of the causal network between pollutants, microbiota, and health outcomes. Interdisciplinary efforts bridging environmental toxicology, microbiology, nutrition, and precision medicine are essential for developing microbiota-based countermeasures against complex environmental challenges. In conclusion, understanding the gut microbiota's role in pollutant toxicity not only deepens our insight into environmental pathophysiology but also opens new avenues for microbiota-targeted prevention, therapy, and policy innovation. Addressing this frontier will be pivotal for safeguarding both human and ecosystem health in an era of escalating environmental pressure.
